# Chromosome-Level Clam Genome Helps Elucidate the Molecular Basis of Adaptation to a Buried Lifestyle

**DOI:** 10.1016/j.isci.2020.101148

**Published:** 2020-05-11

**Authors:** Min Wei, Hongxing Ge, Changwei Shao, Xiwu Yan, Hongtao Nie, Haibao Duan, Xiaoting Liao, Min Zhang, Yihua Chen, Dongdong Zhang, Zhiguo Dong

**Affiliations:** 1 Jiangsu Key Laboratory of Marine Bioresources and Environment, Jiangsu Ocean University, Lianyungang 222005, China; 2 Jiangsu Key Laboratory of Marine Biotechnology, Jiangsu Ocean University, Lianyungang 222005, China; 3 Co-Innovation Center of Jiangsu Marine Bio-industry Technology, Jiangsu Ocean University, Lianyungang 222005, China; 4 College of Fisheries and Life Science, Dalian Ocean University, Dalian 116023, China; 5 Yellow Sea Fisheries Research Institute, Chinese Academy of Fishery Sciences, Qingdao 266071, China

**Keywords:** Biological Sciences, Genomics, Genomic Analysis, Omics

## Abstract

Bivalve mollusks are economically important invertebrates that exhibit marked diversity in benthic lifestyle and provide valuable resources for understanding the molecular basis of adaptation to benthic life. In this report, we present a high-quality, chromosome-anchored reference genome of the Venus clam, *Cyclina sinensis*. The chromosome-level genome was assembled by Pacific Bioscience single-molecule real-time sequencing, Illumina paired-end sequencing, 10× Genomics, and high-throughput chromosome conformation capture technologies. The final genome assembly of *C. sinensis* is 903.2 Mb in size, with a contig N50 size of 2.6 Mb and a scaffold N50 size of 46.5 Mb. Enrichment analyses of significantly expanded and positively selected genes suggested evolutionary adaptation of this clam to buried life. In addition, a change in shell color represents another mechanism of adaptation to burial in sediment. The high-quality genome generated in this work provides a valuable resource for investigating the molecular mechanisms of adaptation to buried lifestyle.

## Highlights

• A chromosome-level assembly for clam genome is provided
• The evolutionary order of bivalve adductor muscle is from double to single
• The work suggests evolutionary adaptations to a buried lifestyle
• Change of shell color represents another mechanism of adaptation to burial in sediment

## Graphical Abstract

**Figure:**
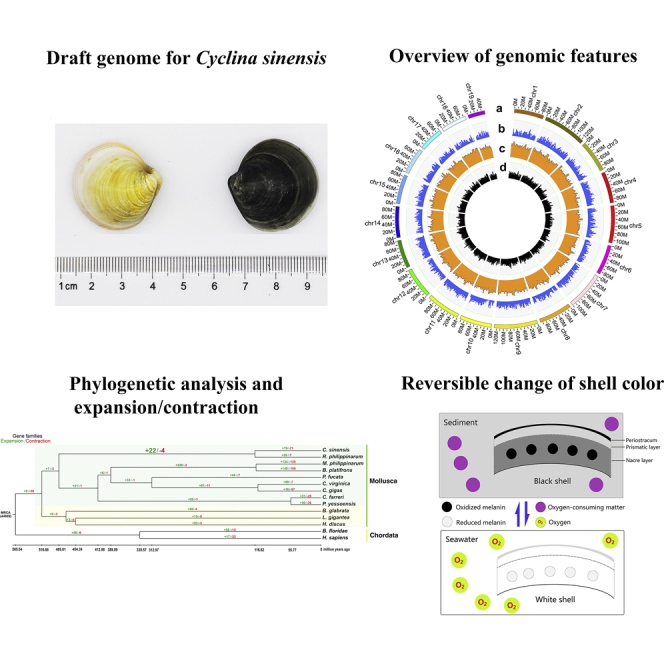

## Introduction

Bivalves are a large superclade of mollusks, consisting of approximate 10,000 species with a global distribution in diverse marine, freshwater, and terrestrial environments (Appeltans et al., 2012). Most bivalves are important fishery and aquaculture species, providing significant economic benefits to humans. Bivalves have undergone little change in lifestyle over 500 million years (Barnosky et al., 2011), including members that are sessile, semisessile, burrowing, or free-living filter feeders. Bivalves are well adapted to benthic life and play critical roles in benthic ecological processes. Among the bivalves, benthic bivalves buried in sediment play important roles in natural biochemical cycles and in material exchange between water and sediment (Vaughn and Hakenkamp, 2001). The sediment microenvironment is especially complex, because it consists of both water and soil, and benthic bivalves have adapted to extreme environments with a low oxygen content, pathogens, and high reducing power (Wang et al., 2012, Costa et al., 2015, Collins et al., 2017, Santos et al., 2019). The most burrowing and buried bivalves play critical roles in bioturbation and the breakdown of organic matter in sediment, improving the sediment microenvironment for the growth of bacteria and protists (Newel, 2004, Norkko and Shumway, 2011). Despite the biological, ecological, and economic significance of these bivalves, available genomes are still limited to a few species (Yan et al., 2019, Ran et al., 2019, Bai et al., 2019), which hinders our understanding of the molecular basis of adaptation to a buried lifestyle in sediment.

Bivalves undergo extraordinary metamorphosis during their life cycle, including the transition from pelagic life (trochophores and veligers) to benthic life (pediveliger larvae) (Yan et al., 2019) and then into lineage-specific benthic lifestyles for juveniles and adults, such as sessile, semisessile, and burrowing lifestyles. For adaptation, lineage-specific biological features are formed, such as differences in the adductor muscle, the foot muscle, and shell shape. The adductor muscle differs greatly in quantity and size between bivalves with different lifestyles. As burrowing bivalves, clams have double adductor muscles and bury themselves in sediment to avoid predation (Yan et al., 2019, Ran et al., 2019, Bai et al., 2019) and are thus significantly different from other lineages of bivalves, such as oysters (Zhang et al., 2012) and scallops (Wang et al., 2017, Li et al., 2017). Oysters have only one posterior adductor muscle and attach their left, larger shell to rocks or other hard surfaces, displaying a sessile lifestyle (Zhang et al., 2012). Scallops also have a large posterior adductor muscle, and most of adductor muscle is striated muscle acting to close the shell quickly, probably as an adaptation to swimming as part of their free-living lifestyle (Guderley and Tremblay, 2016). The Venus clam, *Cyclina sinensis*, is an economically important marine bivalve widely distributed in the coastal muddy sands of China, Korea, Japan, and Southeast Asia (Wang et al., 2005b). This clam possesses a burrowing lifestyle typical of clams, accompanied by two adductor muscles, a muscular foot, and a nearly round shell. Thus, the Venus clam is an excellent organism for studying molecular adaptations to benthic life.

In this study, we report a high-quality, chromosome-anchored reference genome of the Venus clam, *C. sinensis*. The chromosome-level genome of *C. sinensis* was assembled with a combination of whole-genome sequencing (Pacific Biosciences single-molecule real-time sequencing and Illumina paired-end sequencing) and genome mapping (10× Genomics and high-throughput chromosome conformation capture technology) technologies. Comparative genomic analyses of gene expansion, gene contraction, and positive selection on genes among species with different benthic lifestyles were also conducted, helping elucidate the molecular basis of adaptation to a burrowing lifestyle in clams.

## Results

### Genome Sequencing and Assembly

A total of 58.02 Gb of reads (67.2×) with an insert size of 350 bp was obtained with the Illumina HiSeq PE150 platform (see Table S1), and a total of 103.29 Gb of reads (119.6×) was obtained with the PacBio Sequel platform (see Table S2). Two genome mapping technologies, 10× Genomics and high-throughput chromosome conformation capture technologies, were also employed, yielding a total of 123.28 Gb of reads for 10× Genomics data (142.3×) and a total of 102.2 Gb of reads (118.3×) for Hi-C data (see Tables S3 and S4). In total, we obtained 386.8 Gb (447.7×) of raw genome sequence data (see Table S5). In addition, a total of 74.3 Gb of transcriptomic data was obtained for genome annotation (see Table S6).

**Figure 1:**
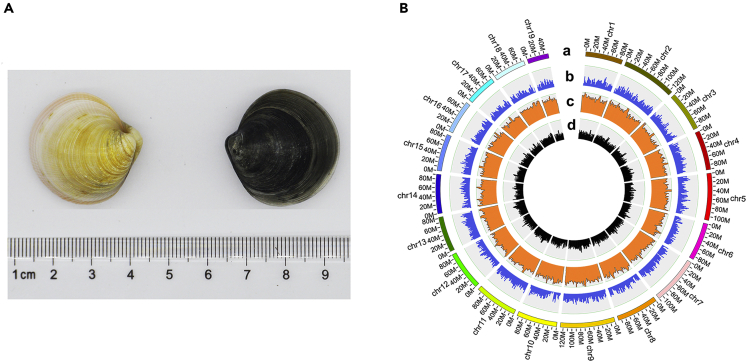
Diagram and Genomic Landscape of the clam *C. sinensis* (A) Two-year-old clams with two shell colors: the light yellow shell represents a clam that was dug out of the sediment and cultured in pool without sediment for a long time, and the black shell represents a clam that was just dug out of the sediment. (B) From outer to inner circles: a represents the 19 haploid chromosomes at the Mb scale; b represents gene density (blue lines) on each chromosome; c represents repeat density (orange lines) across the genome; and d represents GC content, drawn in 2-Mb sliding windows.

Prior to *C. sinensis* genome assembly, 58.02 Gb of Illumina data was used to estimate genome size (864 Mb) and genome heterozygosity (1.53%) based on *k*-mer analysis (see Table S7). After contig assembly procedures, error-corrected and high-quality assembled contigs were finally obtained using PacBio platform data, and the total length of the assembled contigs was 902.8 Mb, with a contig N50 size of 2.6 Mb (see Table S8). In addition, two assisting assembly technologies were employed to produce the final assembled genome (see Table S9). The final genome assembly was 903.2 Mb in length (total length of scaffolds), with a contig N50 size of 2.6 Mb, a scaffold N50 size of 46.5 Mb and assigned to the 19 haploid chromosomes (see Table S10 and Figure 1), representing significant improvements over most published bivalve genomes (contig N50 sizes of 1.6 kb–1.79 Mb, scaffold N50 sizes of 14.5 kb–75.94 Mb; see Table S11) (Zhang et al., 2012, Takeuchi et al., 2012, Takeuchi et al., 2016, Wang et al., 2017, Sun et al., 2017, Yan et al., 2019, Li et al., 2017, Li et al., 2018, Ran et al., 2019, Uliano-Silva et al., 2018, Gómez-Chiarri et al., 2015, Powell et al., 2018, Mun et al., 2017, Bai et al., 2019).

The 95.59% read mapping rate, 99.8% genome coverage rate of reads (see Table S12), 0.81% heterozygous SNP rate and 0.0008% homologous SNP rate (see Table S13) of the final assembled genome verified its consistency and completeness. A total of 232 Core Eukaryotic Genes Mapping Approach (CEGMA) identified core genes with 93.55% completeness (see Table S14), together with 92.7% complete and 1.3% fragmented Benchmarking Universal Single-Copy Orthologs (BUSCO) (see Table S15), were identified in the final assembled genome, indicating the high degree of completeness of the gene regions.

### Genome Annotation

Tandem repeats and transposable elements (TEs) were identified in the assembled *C. sinensis* genome. The repeat content accounted for 43.14% (389.6 Mb) of the assembled genome (see Table S16). Within this repeat content, TEs accounted for 36.01% of the genome (see Table S17), with 23.58% accounted for by DNA transposons and 12.43% accounted for by retrotransposons (5.23% long interspersed nuclear elements, 0.28% short interspersed nuclear elements, and 6.92% long terminal repeats), and showed high divergence (see Figure S2). Noncoding RNA (ncRNA) genes (transfer RNAs, ribosomal RNAs, microRNAs, and small nuclear RNAs) were also predicted, and a total of 0.31 Mb of ncRNAs was predicted in the *de novo*-assembled *C. sinensis* genome, accounting for 43.14% of the genome (see Table S18). With gene prediction and functional annotation, a final nonredundant consensus gene set for *C. sinensis* was obtained, and 27,564 protein-coding genes were predicted in the final assembled genome (see Table S19 and Figure S3), which is similar to the number in other published bivalve genomes (see Table S20 and Figure S4). Finally, 27,344 protein-coding genes were annotated, accounting for 99.2% of all the predicted genes (see Table S21).

### Gene Family Analysis

**Figure 2:**
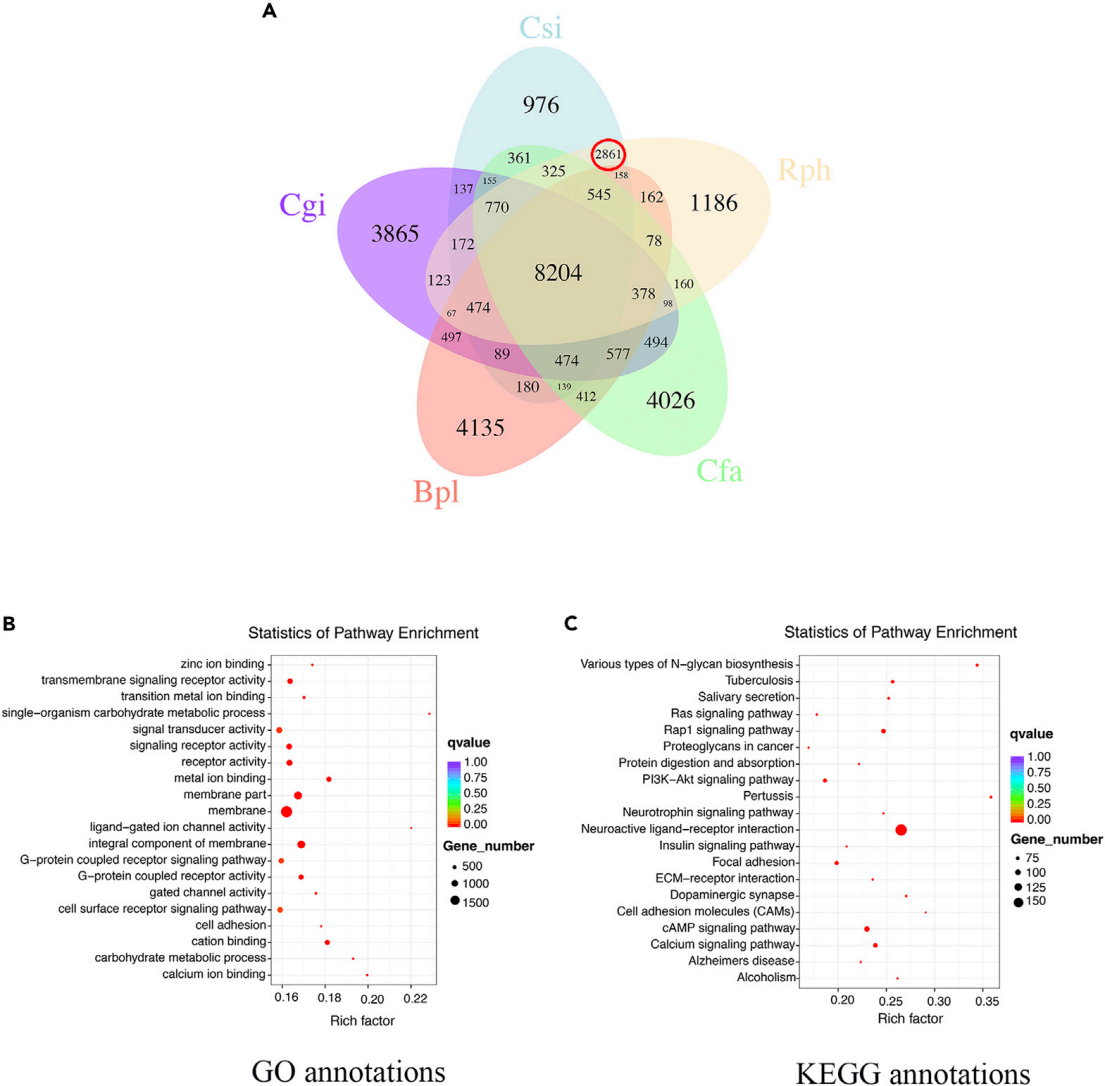
Venn Diagram of Gene Families among Five Bivalves and Enrichment Analysis of Gene Families Specific to Two Buried Bivalves (*C. sinensis* and *R. philippinarum*) (A) Common and unique gene families among five bivalves shown with a Venn diagram: Csi, *C. sinensis*; Rph, *R. philippinarum*; Cfa, *C. farreri*; Bpl, *B. platifrons*; Cgi, *C. gigas*. The number in the red circle represents the number of gene families specific to the two buried bivalves (*C. sinensis* and *R. philippinarum*). (B) Gene Ontology (GO) enrichment analysis of gene families specific to the two buried bivalves. (C) Kyoto Encyclopedia of Genes and Genomes (KEGG) enrichment analysis of gene families specific to the two buried bivalves. The enrichment factor represents the degree of enrichment, with a larger value indicating a greater degree of enrichment. The solid circle represents the GO term or KEGG pathway in which the specific gene families are enriched, and the larger the solid circle, the more gene families it contains. The q value was obtained by correction of the p value of the GO term or KEGG pathway for multiple comparisons. The color of the solid circle represents the q value, with deeper red indicating a smaller q value and stronger enrichment.

Gene families were defined among 14 selected species (12 mollusk species) in the present study. In total, 44,679 gene families and 325 shared single-copy gene families were identified in the 14 selected species (see Table S22 and Figure S5). Gene families present in *C. sinensis* but not in any other species were regarded as *C. sinensis*-specific gene families, and a total of 601 gene families presented exclusively in *C. sinensis* compared with the other 13 selected species were associated with in 25 Gene Ontology (GO) terms and enriched in 29 Kyoto Encyclopedia of Genes and Genomes (KEGG) pathways (see Tables S23 and S24; Figure 2A). Moreover, 2,861 gene families were identified as specific to two buried bivalves (*C. sinensis* and *Ruditapes philippinarum*) compared with three sessile/semisessile bivalves (*Chlamys farreri*, *Crassostrea gigas*, and *Bathymodiolus platifrons*) (Figure 2A). The buried bivalve-specific gene families were enriched in 107 GO terms and 80 KEGG pathways (see Tables S25, S26, and S27; Figure 2B), mainly in association with a number of complex signaling systems (such as PI3K-Akt, Ras, Rap1, cAMP signaling, and calcium signaling pathways), ion binding (such as “zinc ion,” “transition metal ion,” “metal ion,” “cation,” and “calcium ion binding”), and the immune system (such as “*Staphylococcus aureus* infection,” “inflammatory mediator regulation of TRP channels,” and “salivary secretion”) (see Table S28).

### Genome Evolution and Evolutionary Rate Estimation

To investigate the phylogenetic evolutionary relationships of *C. sinensis* with other species, a phylogenetic tree was reconstructed based on 325 shared single-copy gene families retrieved from the above gene family analysis (*Homo sapiens* and *Branchiostoma floridae* were chosen as the outgroup species). Phylogenetic analysis suggested that *C. sinensis* diverged from *R. philippinarum* approximately 122 million years ago (mya). The clam lineage diverged from the bivalve lineage approximately 485 mya, and Bivalvia showed an estimated time of divergence from its sister group Gastropoda of approximately 516 mya (see Figure S6).

In the analysis of positive/negative selection on genes, nine positively selected genes were detected among the genes shared by the two buried bivalves (see Table S29), and GO and KEGG enrichment analyses of the positively selected genes revealed that they were enriched in 19 GO terms and 6 KEGG pathways (see Tables S30 and S31), mainly in association with regulation of metal ion transport (*nkain3*) (Gorokhova et al., 2007), immune response (*fbxl2* and *yipf4*) (Chen et al., 2013, Müller et al., 2015), cellular proliferation (*caprin-1*) (Wang et al., 2005a), formation and maintenance of skeletal muscle (actn) (Yang et al., 2009), and RNA processing (*mthfsd*) (MacNair et al., 2016).

### Expansion and Contraction of Gene Families

**Figure 3:**
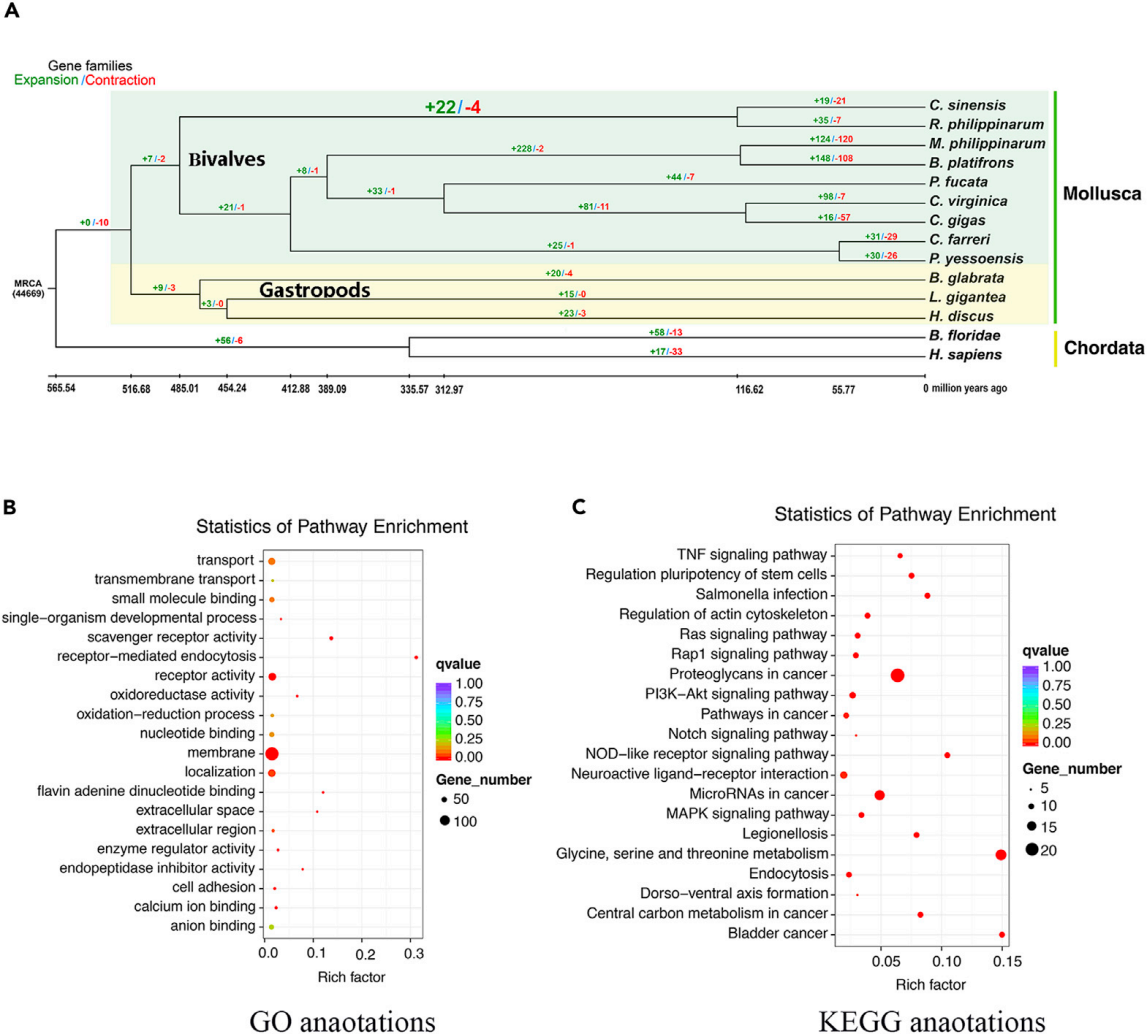
Phylogenetic Analysis of *C. sinensis* and Enrichment Analysis of Expanded Gene Families in Two Buried Bivalves (*C. sinensis* and *R. philippinarum*) (A) A phylogenetic tree was constructed based on 325 shared single-copy gene families retrieved from 14 selected species. *H. sapiens* and *B. floridae* were chosen as the outgroup species. The green and red numbers on the branches represent the expanded and contracted gene families, respectively. The green and red numbers in the red frame represent the expanded and contracted gene families in two buried bivalves (*C. sinensis* and *R. philippinarum*). (B) GO enrichment analysis of expanded gene families in the two buried bivalves. (C) KEGG enrichment analysis of expanded gene families in the two buried bivalves.

After further screening, 44,669 gene families of the most recent common ancestor were used in an analysis of expansion and contraction. Compared with *R. philippinarum*, 19 expanded and 21 contracted gene families were detected in *C. sinensis* (see Figure 3A), and the expanded genes in *C. sinensis* were enriched in 56 GO terms and 22 KEGG pathways (see Tables S32 and S33). Moreover, compared with seven sessile/semisessile bivalves (*Modiolus philippinarum*, *B. platifrons*, *Pinctada fucata martensii*, *Crassostrea virginica*, *C. gigas*, *C. farreri*, and *Patinopecten yessoensis*), 24 expanded gene families (4 contracted gene families) were detected in the two buried bivalves (*R. philippinarum* and *C. sinensis*) (see Figure 3B; Table S34). Enrichment analyses of the expanded genes revealed that they were enriched in 40 GO terms and 20 KEGG pathways (see Figure 3B; Tables S35 and S36), primarily in association with immune systems (such as “proteoglycans in cancer,” “scavenger receptor activity,” “salmonella infection,” “TNF signaling pathway,” and “PI3K-Akt signaling pathway”; see Table S37) and redox processes (such as “oxidoreductase activity,” “oxidation-reduction process,” and “flavin adenine dinucleotide binding”; see Table S38), indicative of adaptation to burial in sediment environments. A number of immune-related genes were expanded in two buried bivalves, including interferon-inducible GTPase 5 (*Iigp5*) and heat shock protein 70 (Hsp70) member 12 (*Hsp70_12*), and they were enriched in “TNF signaling pathway” and “proteoglycans in cancer,” respectively. In addition, the expansion genes (glucose dehydrogenases, *GDHs*) of FAD- or PQQ-dependent GDH family in two buried bivalves were enriched in “oxidoreductase activity,” “oxidation-reduction process,” and “flavin adenine dinucleotide binding.”

### Observation of Color Change and Melanin in Shells

*C. sinensis* displays a variety of shell colors, such as black, white, brownish yellow, and purple. An interesting phenomenon is observed: the shell color changes from black to white or brownish yellow are reversible under different environmental conditions (in and out of mud) (see Figure S7). In addition, the black shells of living clams show the same time course of fading as dissected black shells. To observe the color distribution, the black shell of *C. sinensis* individuals were cut and observed under a stereomicroscope. The results showed that the black color was mainly found in the nacre layer and periostracum of the shell (see Figure S8). To identify the black matter, black pigment isolated from the black clam shells was dissolved in 0.01 mol/L sodium hydroxide solution and identified by UV spectral scanning. The results showed two major absorption peaks at 213 and 280 nm (see Figure S9), which share similar characteristic peaks of melanin (Lin et al., 2005, Hao et al., 2015). Moreover, a tyrosinase gene family was detected in the buried bivalve-specific gene families (see Table S39), and the tyrosinase genes were enriched in “melanogenesis,” “betalain biosynthesis,” and “riboflavin metabolism.”

## Discussion

Bivalves are a fascinating group of animals that are well adapted to benthic life and play critical roles in maintaining the diversity of benthic ecology. To adapt to complex and diverse benthic environments, bivalves have evolved a variety of benthic lifestyles. For adaptation, lineage-specific biological features have evolved in bivalves, especially differences in the adductor muscle. Interestingly, most bivalves with single adductor muscles are adapted to sessile and semisessile benthic lifestyles, such as oysters (Zhang et al., 2012) and scallops (Wang et al., 2017, Li et al., 2017). Most bivalves with double adductors are adapted to buried lifestyles, such as the Venus clam (*C. sinensis*), the Manila clam (*R. philippinarum*) (Yan et al., 2019, Mun et al., 2017), the blood clam (*Scapharca broughtonii*) (Bai et al., 2019), and the razor clam (*Sinonovacula constricta*) (Ran et al., 2019). There seem to be obvious correlations between the features of the adductor muscle and a benthic lifestyle, and the double-adductor morphology is more suitable than others for a buried lifestyle.

*C. sinensis* and *R. philippinarum* are typical buried bivalves with double adductor muscles and are closely phylogenetically related (see Figure 3A). In the phylogenetic analysis performed at the genomic level, the double-adductor buried bivalves (~485 mya) differentiated earlier than the single-adductor or sessile/semisessile bivalves (~516 mya) (see Figure S6), supported by the phylogenetic position of the razor clam (Ran et al., 2019). The sediment microenvironment is extremely complex, as it consists of both water and soil, and benthic bivalves are adapted to extreme environments with a low oxygen content, enriched ions, and enriched pathogens (Wang et al., 2012, Costa et al., 2015, Collins et al., 2017, Santos et al., 2019). Therefore, the existence of specific molecular mechanisms underlying the tolerance of extreme environments in benthic bivalves seems likely. The gene families specific to bivalves with buried lifestyles that are involved in complex signaling systems, ion binding systems, and the immune system play important roles in adaptation to burial in sediment.

Expansion of gene families plays the most important role in phenotypic diversity and evolutionary adaptation to the environment (Rayna and Hans, 2015). Most shellfish possess the innate immune system and lack an adaptive immune system. Interferon-inducible GTPases are expressed in host cells by induction of interferons and involved in host innate defense via regulation of pathogen degradation in host cells (Taylor, 2007). Most heat shock proteins (Hsps) are generally stress inducible as they play a particularly important cytoprotective role in cells exposed to stressful conditions, and Hsp70 is involved in stimulation of both the innate and adaptive immune systems (Zininga et al., 2018). It also participates in the multistress resistance and has potential roles in the immune responses of *R. philippinarum* (Yan et al., 2019). Overall, the expansion genes (*Iigp5* and *Hsp70*) of interferon-inducible GTPase and Hsp70 families in buried bivalves are vital to the resistance to pathogen-rich and hypoxia burial conditions and the buried adaptation of buried bivalves. In addition to immune systems, the expanded gene families in the two buried bivalves are mainly involved in a special physiological process, the redox process (see Figure 3B). The large amount of oxygen-consuming organic matter and low oxygen content in buried sediment make it an environment with high reducing power (Collins et al., 2017), which suggests that these expanded gene families enriched in redox processes play a vital role in adaptation to burial in sediment with high reducing power. Glucose oxidoreductases, enzymes catalyzing the oxidation of glucose, can be divided into two major groups based on their electron acceptors: glucose oxygen-oxidoreductase (GOD) and glucose dehydrogenases (GDHs). GOD catalyzes the oxidation of glucose using molecular oxygen as the electron acceptor and is limited by dissolved oxygen concentration. GDHs can participate in the oxidation of glucose using nicotine adenine dinucleotide (NAD), nicotine adenine dinucleotide phosphate (NADP), pyrroloquinoline quinone (PQQ), or flavin adenine dinucleotide (FAD) as an electron acceptor without the consumption of oxygen (Tsachaki et al., 2018, Okuda-Shimazaki et al., 2020). Therefore, because they were detected among the expanded gene families, FAD- or PQQ-dependent GDHs may play a vital role in adaptation to a buried lifestyle at low oxygen concentrations.

**Figure 4:**
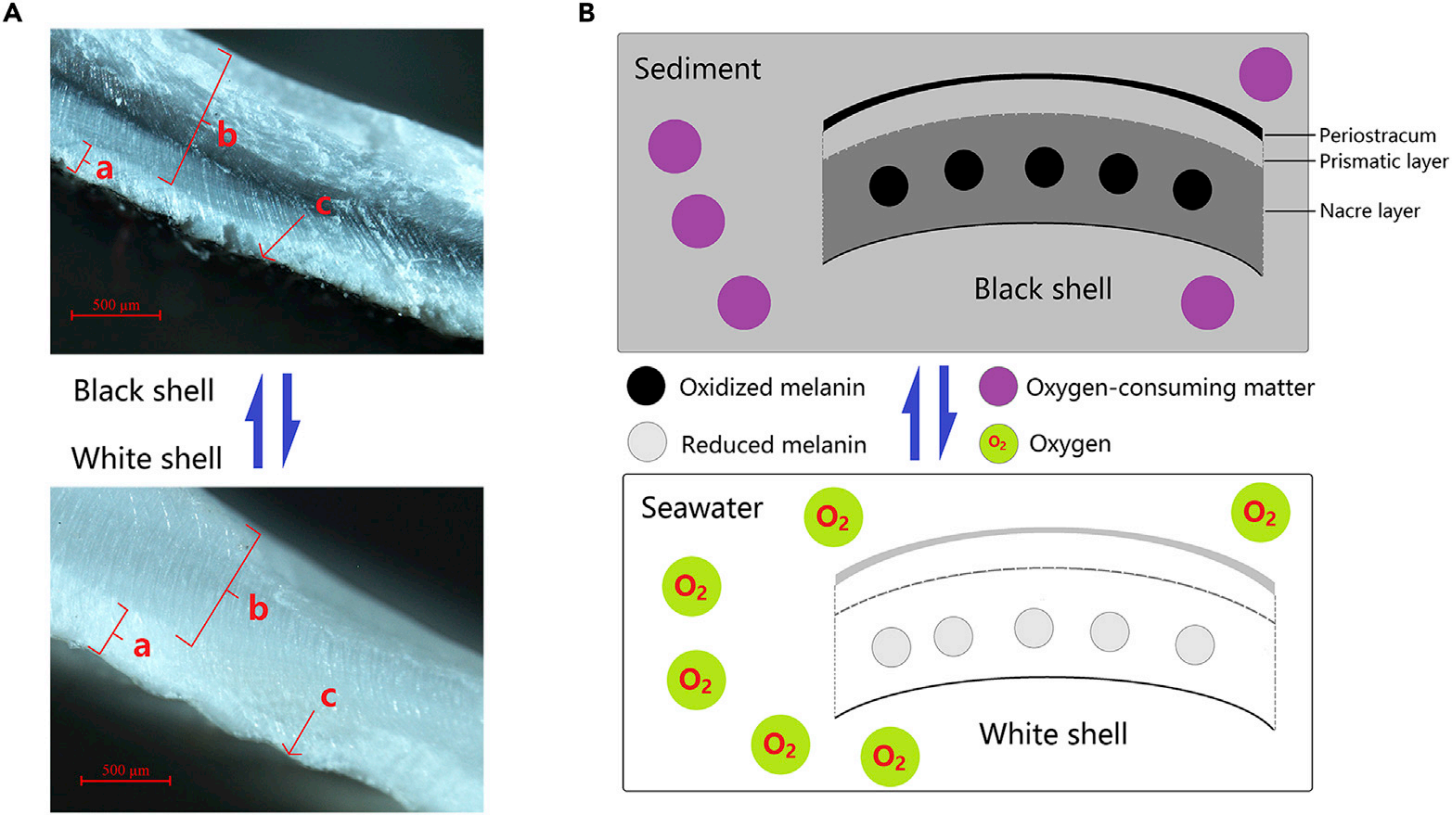
Reversible Change in Clam Shell Color in Different Environments (A) Cross-section of a black and white shell of *C. sinensis* at high magnification (8×) under a stereomicroscope; a represents the prismatic layer in the clam shell; b represents the nacre layer; c represents the periostracum. (B) Schematic representation of the reversible change in clam shell color in different environments (buried in sediment and cultured in ponds without sediment). The blue two-way arrow indicates that the change in clam shell color is reversible.

Interestingly, color changes (fading from black to white or brownish yellow) in the shell of clams under different environmental conditions (in or out of muddy sediment) are reversible, probably owing to melanin changes in the shell. Melanin possesses redox activity and can be repeatedly switched between oxidized and reduced states, and antioxidant activities are insensitive to its redox state (Kim et al., 2014), indicating that the black color of the shell is due to the reduction of melanin in the shell by the high-reducing-power sediment environment and that the fading of black shells is due to the oxidation of melanin by oxygen in air or seawater (see Figure 4). The melanin in the shell can be repeatedly switched between oxidized and reduced states by the environment and consequently lead to changes in shell color for simulating the environment color, which represents another mechanism of adaptation to different environments, especially adaptation to burial in sediment for avoiding predation. Moreover, the tyrosinase gene family, which plays a key role in the synthesis of melanin, was specific to the two buried bivalves studied here (see Table S39; Yokoyama et al., 1990, Koga et al., 1999), which provides a molecular basis for the adaptation to burial.

In conclusion, we obtained a high-quality chromosome-level genome assembly of *C. sinensis* in the present study. The clam genome was 903.2 Mb in size, with a contig N50 size of 2.6 Mb, a scaffold N50 size of 46.5 Mb, and anchored into the 19 haploid chromosomes. Enrichment analyses of the expanded and unique gene families in two buried bivalves suggested the evolutionary adaptation of bivalves to a buried lifestyle. The expansion genes (*Iigp5*, *Hsp70* and *GDH*) and changes in black shell color may play a vital role in adaptation to burial in sediment. Moreover, the obtained genome considerably improves our understanding of the genetics of bivalves and will facilitate further comparative evolutionary research.

### Limitations of the Study

In this report, we present a high-quality chromosome-anchored reference genome of the Venus clam, *C. sinensis*, and provide a comprehensive framework for understanding the genetic adaptations of two bivalves (*C. sinensis* and *R. philippinarum*) to buried life. The high-quality published genomes of buried bivalves are limited to several species, including *R. philippinarum*, *S. broughtonii*, and *S. constricta*. With the development of high-throughput sequencing technology and reduced sequencing costs, more genomes of bivalves will be sequenced and available in the future, which will advance our understanding of the molecular basis of adaptation to a buried lifestyle in benthic bivalves. Functional experimental assays are also required to confirm the expansion genes (*Iigp5*, *Hsp70* and *GDH*) in the two buried bivalves and to identify more targets involved in the adaptation of bivalves to a buried lifestyle. Moreover, more evidence is required to confirm the direct relationship between changes in black shell color and the redox states of melanin in the shell.

### Resource Availability

#### Lead Contact

Further information and requests for resources should be directed to and will be fulfilled by the Lead Contact, Zhiguo Dong (dzg7712@163.com).

#### Materials Availability

This study did not generate new unique reagents.

#### Data and Code Availability

The clam genome assembly reported in this paper has been approved and given the accession number GenBank: JAAONU000000000 under the project PRJNA612143. The genome annotations are also available from the Dryad Digital Repository at https://doi.org/10.5061/dryad.44j0zpcb5.

## Methods

All methods can be found in the accompanying Transparent Methods supplemental file.

## Supplemental Information

Document S1. Transparent Methods, Figures S1–S9, and Tables S1–S24, S29–S33, and S39

Table S25. List of Genes Unique to Two Buried Bivalves (*C. sinensis* and *R. philippinarum*) Compared with Three Bivalves with Other Lifestyles (*C. farreri*, *C. gigas* and *B. platifrons*), Related to Figure 2

Table S26. GO Enrichment of Unique Gene Families in Two Buried Bivalves (*C. sinensis* and *R. philippinarum*) Compared with Three Bivalves with Other Lifestyles (*C. farreri*, *C. gigas* and *B. platifrons*), Related to Figure 2

Table S27. KEGG Enrichment of Unique Gene Families in Two Buried Bivalves (*C. sinensis* and *R. philippinarum*) Compared with Three Bivalves with Other Lifestyles (*C. farreri*, *C. gigas* and *B. platifrons*), Related to Figure 2

Table S28. Unique Gene Families Enriched in the Key Physiological Process in Two Buried Bivalves (*C. sinensis* and *R. philippinarum*) Compared with Three Bivalves with Other Lifestyles (*C. farreri*, *C. gigas* and *B. platifrons*), Related to Figure 2

Table S34. List of Genes in 24 Expanded Gene Families in Two Buried Bivalves (*C. sinensis* and *R. philippinarum*), Related to Figure 3

Table S35. GO Enrichment of Expanded Gene Families in Two Buried Bivalves (*C. sinensis* and *R. philippinarum*), Related to Figure 3

Table S36. KEGG Enrichment of Expanded Gene Families in Two Buried Bivalves (*C. sinensis* and *R. philippinarum*), Related to Figure 3

Table S37. List of Genes in Expanded Gene Families Enriched in the Immune Systems in Two Buried Bivalves (*C. sinensis* and *R. philippinarum*), Related to Figure 3

Table S38. List of Genes in Expanded Gene Families Enriched in the Redox Process in Two Buried Bivalves (*C. sinensis* and *R. philippinarum*), Related to Figure 3
