# Supplementary material for: Chromosome-Level Clam Genome Helps Elucidate the Molecular Basis of Adaptation to a Buried Lifestyle
Source: iScience. 2020 May 11;23(6):101148. doi: 10.1016/j.isci.2020.101148 (PMC7251785; doi:10.1016/j.isci.2020.101148)
Supplement: Document S1. Transparent Methods, Figures S1–S9, and Tables S1–S24, S29–S33, and S39 [file mmc1.pdf]

iScience, Volume *23*

Supplemental Information

Chromosome-Level Clam Genome Helps Elucidate the Molecular Basis of Adaptation to a Buried Lifestyle

Min Wei, Hongxing Ge, Changwei Shao, Xiwu Yan, Hongtao Nie, Haibao Duan, Xiaoting Liao, Min Zhang, Yihua Chen, Dongdong Zhang, and Zhiguo Dong

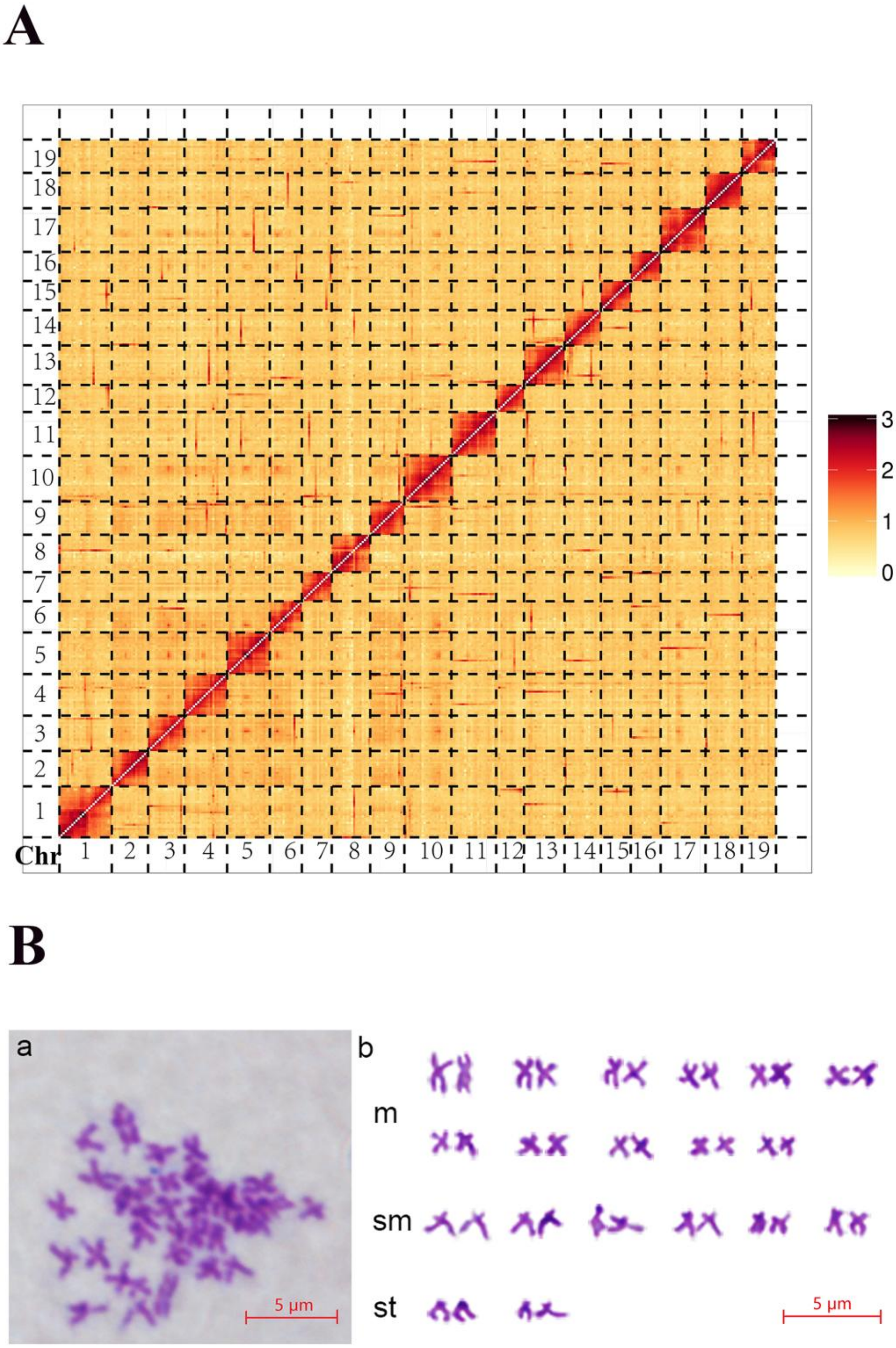

Figure S1. Chromosomal contact maps of *C. sinensis*. (A) Chromosomal contact maps using Hi-C data. The blocks refer to the contacts between one location and another. The deeper colors represent the higher intensity of contact. **(B)** Chromosome

karyotype of *C. sinensis*. m: metacentric chromosome; sm: submetacentric chromosome; st: proximal centromere chromosome. Related to Figure 1.

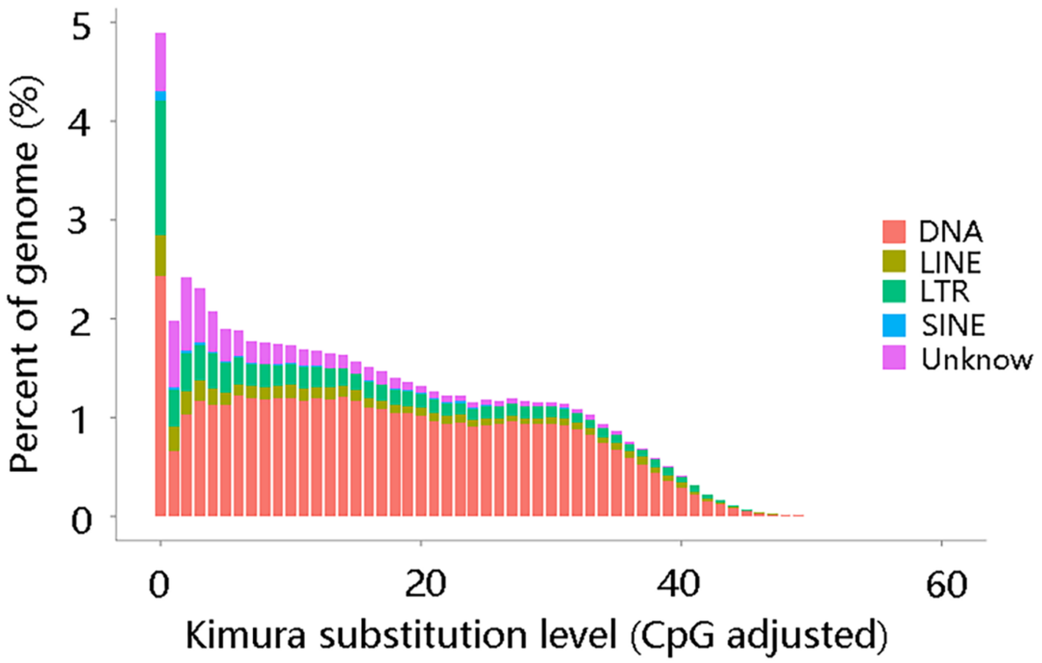

Figure S2. Divergence distribution of transposable elements (TEs) in the *C. sinensis* genome. DNA represents a DNA transposon and is shown in red; LINE represents a long interspersed nuclear element and is shown in dark yellow; LTR represents a long terminal repeat and is shown in green; SINE represents a short interspersed nuclear element and is shown in light blue; unknown TEs are shown in purple. Related to Figure 1.

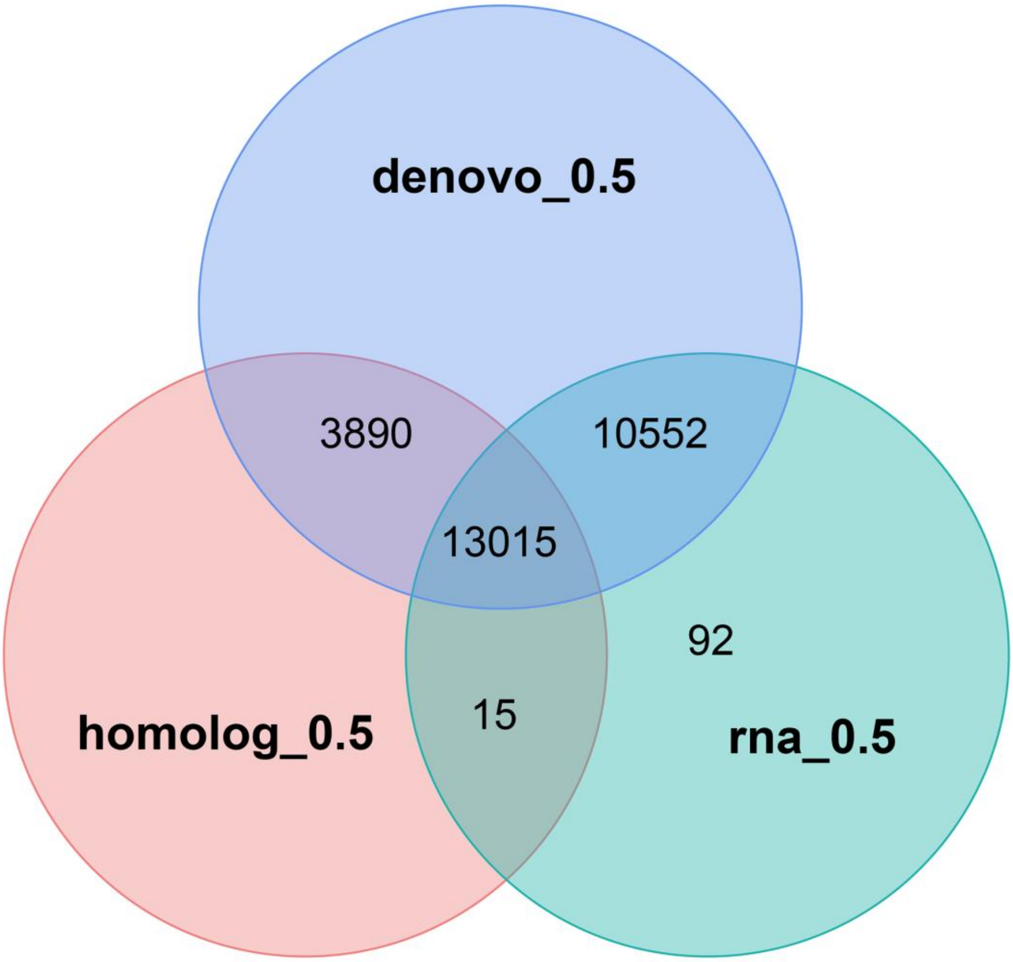

Figure S3. Evidence supports the use of gene sets based on three approaches. The prediction of genes in the *C. sinensis* genome was performed using a combination of three approaches, homolog-based, *de novo*, and transcriptome-based predictions. Related to Figure 1.

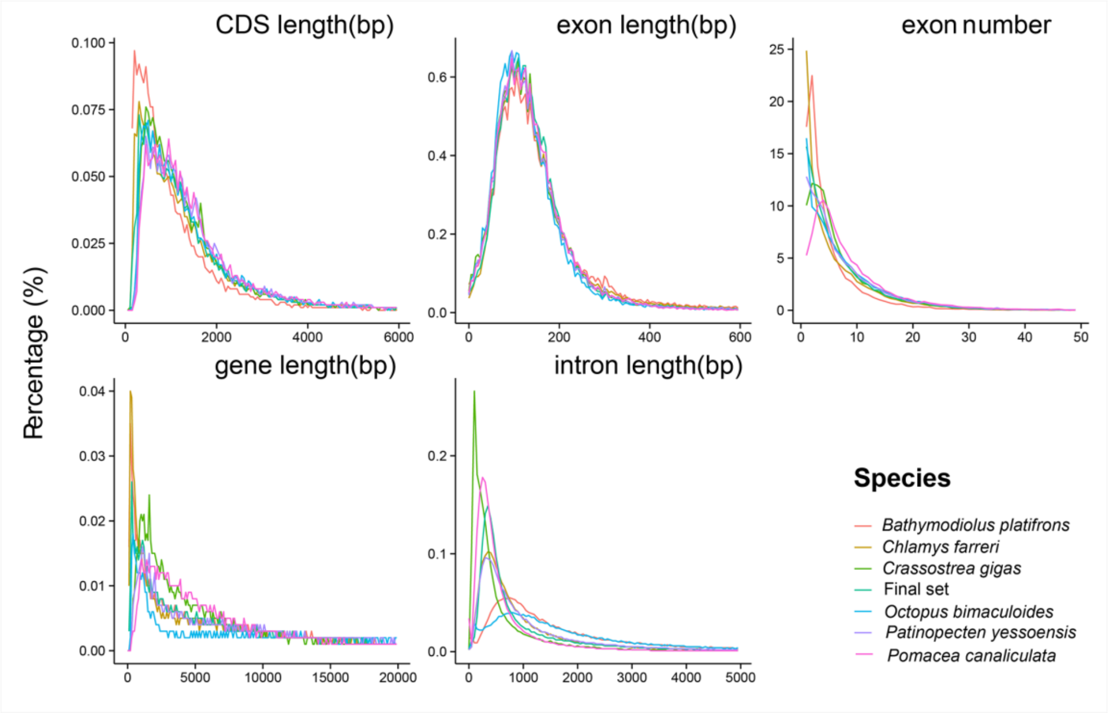

Figure S4. Comparison of gene structure characterization among *C. sinensis* and the other 6 mollusks. The lines with different colors represent different species: the light red line represents *B. platifrons*; the dark yellow line represents *C. farreri*; the green line represents *C. gigas*; the light green line represents *C. sinensis* and is shown with the ‘final set’; the light blue line represents *O. bimaculoides*; the purple line represents *P. yessoensis*; and the pink line represents *Pomacea canaliculata*. Related to Figure 1.

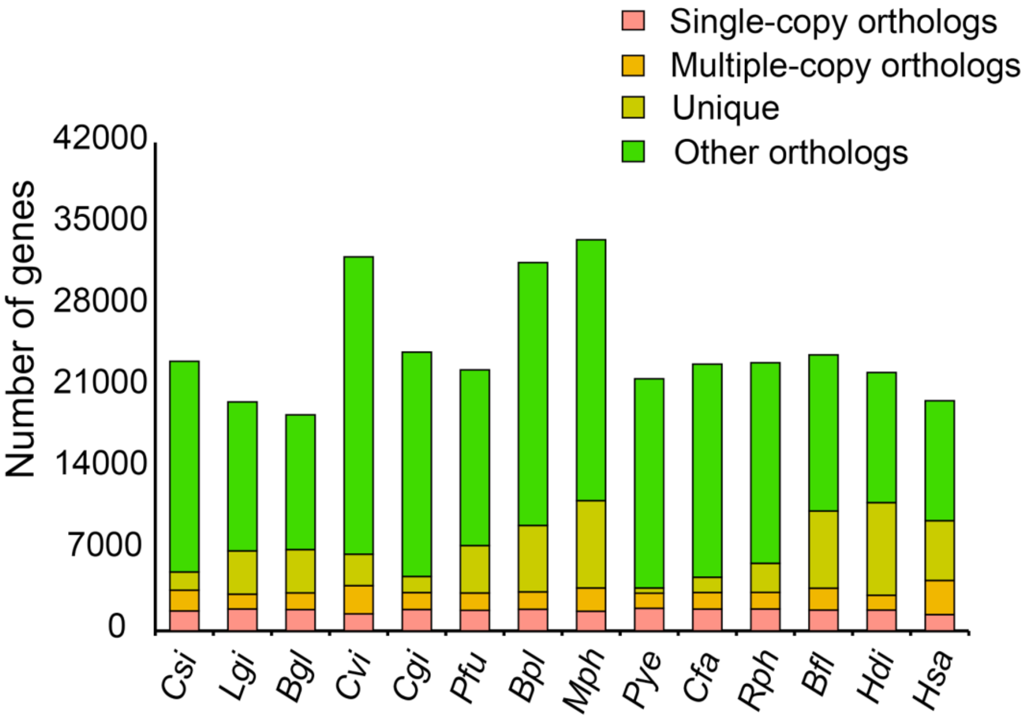

Figure S5. Distribution of genes in 14 different species. Csi, *C. sinensis*, Lgi, *L. gigantea*, Bgl, *B. glabrata*, Cvi, *C. virginica*, Cgi, *C. gigas*, Pfu, *P. fucata*, Bpl, *B. platifrons*, Mph, *M. philippinarum*, Pye, *P. yessoensis*, Cfa, *C. farreri*, Bfl, *B. floridae*, Rph, *R. philippinarum*, Hdi, *H. discus*, Hsa, *H. sapiens*. Different colors represent different types of gene families: pink represents single-copy orthologs; yellow represents multiple-copy orthologs; dark yellow represents unique genes; green represents other orthologs. Related to Figure 1.

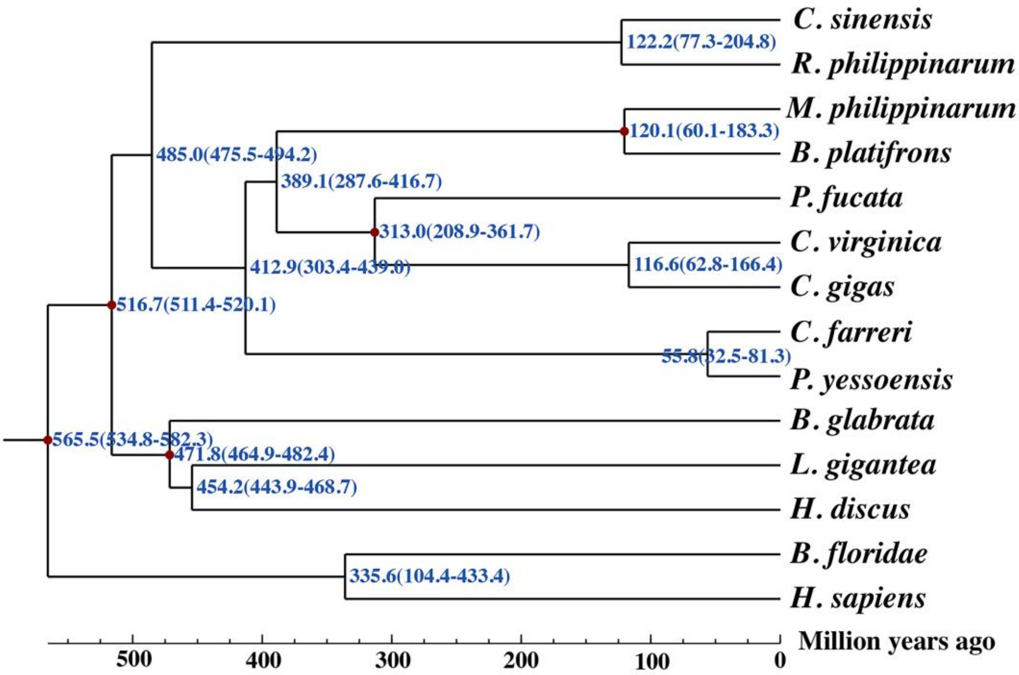

Figure S6. Genome evolution analysis. A phylogenetic tree was constructed based on 325 shared single-copy gene families retrieved from 14 selected species. The five red dots on the branch junctions represent five reference divergence times for calibrations retrieved from the TimeTree database, including divergence times of *B. glabrata* and *H. hannai*, *L. gigantean* and *C. gigas*, *C. gigas* and *P. martensii*, *B. floridae* and *M. philippinarum*, *M. philippinarum* and *B. platifrons*. The blue numbers on the branches represent the estimated diverge times. The split of two buried bivalves (*C. sinensis* and *R. philippinarum*) was estimated at ~485 million years ago. Related to Figure 3.

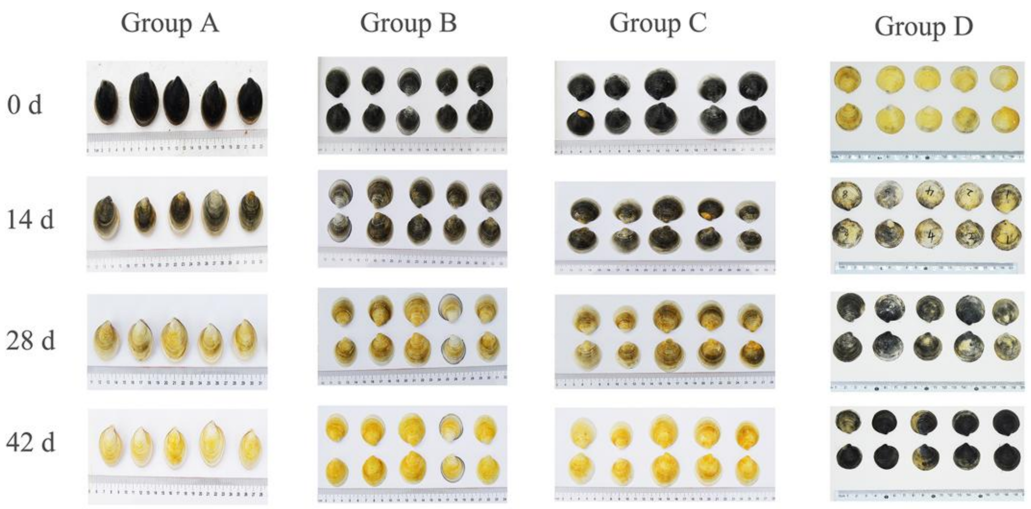

Figure S7. Changes in the black shell color of *C. sinensis* over time. Group A represents the living black-shell clams cultured in seawater. Group B represents the black shells in seawater. Group C represents the black shells in air. Group D represents the white shells in soil. Related to Figure 4.

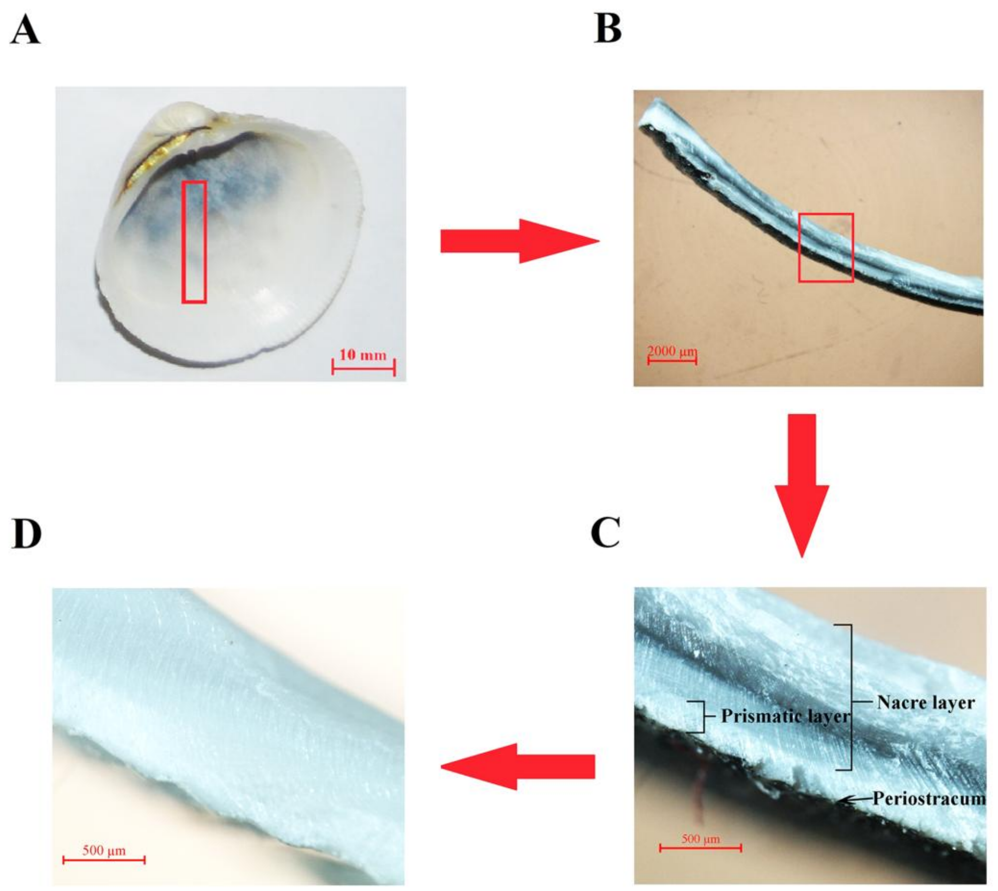

Figure S8. Color distribution in clam shell. (**A**) Black shell of *C. sinensis*. (**B**) Cross-section of the red-framed area in (**A**) at low magnification (0.75×). (**C-D**) Red framed area in (**B**) at high magnification (8×). (**D**) Faded black shell in (**C**). Related to Figure 4.

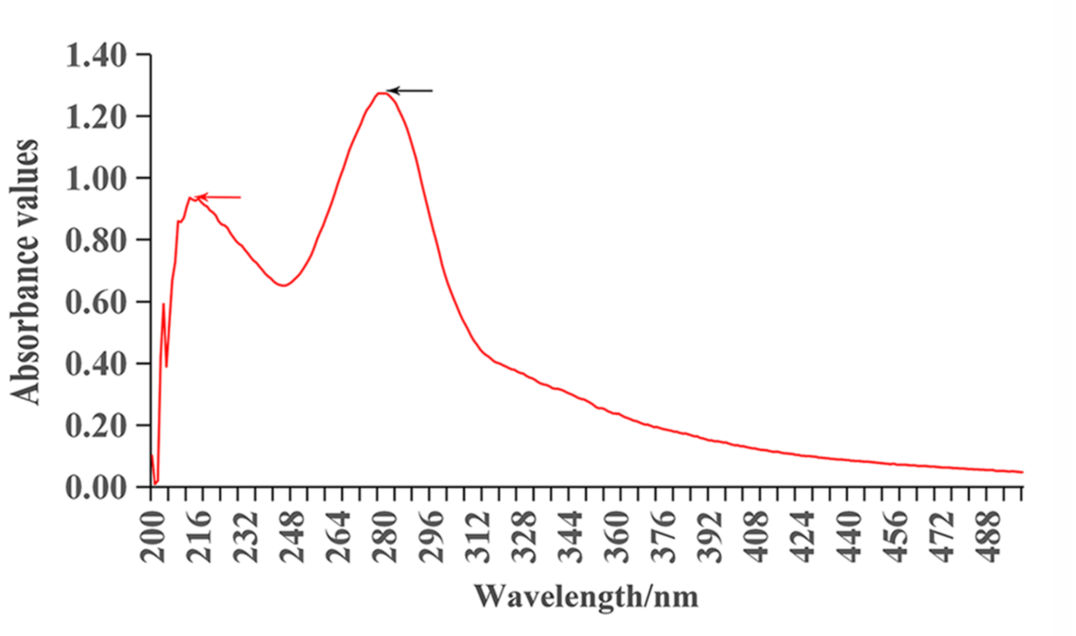

Figure S9. UV spectrum of melanin in black shell. The red and black arrows indicated two main absorption peaks of melanin extracted from the shell. Three replicates were conducted for each sample. Related to Figure 4.

Table S1. Illumina statistics of the genome sequencing data of *C. sinensis*. Related to Figure 1.

Library	Insert size	Raw base (Mb)	Effective rate (%)	Clean base (Mb)	Error rate (%)	Q20 (%)	Q30 (%)	GC (%)
NDES00175_L4	350	37,302	99.89	37,261	0.02	97.44	94.01	35.13
NDES00175_L5	350	20,784	99.89	20,761	0.02	97.71	94.62	35.40

Table S2. PacBio statistics of the genome sequencing data of *C. sinensis*. Related to Figure 1.

Read type	Read base (bp)	Read number	Read length (max)	Read length (mean)	Read length (N50)
Polymerase	103,550,157,654	7,339,298	171,609	14,109	22,575
Insert size	78,156,186,041	7,339,298	131,249	10,649	15,821
Subreads	103,150,157,654	11,679,139	131,249	8,832	13,635

Table S3. 10X Genomics statistics of the genome sequencing data of *C. sinensis*. Related to Figure 1.

Sample name	Raw paired reads	Raw base (Mb)	Effective rate (%)	Error rate (%)*	Q20 (%)*	Q30 (%)*	GC (%)*
NDHX00262-AK138_L3	83,952,972	25,186	97.95	0.02; 0.04	96.59; 91.33	92.28; 83.84	38.70; 37.02
NDHX00262-AK138_L5	3,169,496	951	97.82	0.02; 0.05	96.61; 90.70	92.48; 82.88	38.79; 37.34
NDHX00262-AK139_L3	91,713,547	27,514	97.99	0.02; 0.04	96.55; 91.14	92.21; 83.52	38.70; 37.02
NDHX00262-AK140_L5	4,768,716	1,431	97.99	0.02; 0.05	96.60; 90.67	92.43; 82.81	38.81; 37.37
NDHX00262-AK140_L3	124,029,318	37,209	98.08	0.02; 0.04	96.58; 91.35	92.25; 83.84	38.72; 37.03
NDHX00262-AK139_L5	3,466,318	1,040	97.88	0.02; 0.05	96.62; 90.54	92.49; 82.61	38.79; 37.34
NDHX00262-AK137_L3	96,205,567	28,862	98.11	0.02; 0.04	96.51; 91.42	92.10; 83.93	38.77; 37.11
NDHX00262-AK137_L5	3,612,199	1,084	98.01	0.02; 0.04	96.64; 90.93	92.51; 83.20	38.84; 37.39

Note: * data of two groups represent the analysis results of reads sequenced two times.

Table S4. Hi-c statistics of the genome sequencing data of *C. sinensis*. Related to Figure 1.

Sample name	Raw paired reads (bp)	Raw base (bp)	Effective rate (%)	Error rate (%)*	Q20 (%)*	Q30 (%)*	GC (%)*
RHC00873_L8	10,056,027	3,016,808,100	99.62	0.02; 0.03	98.64; 95.42	96.17; 90.24	35.12; 35.30
RHC00873_L6	84,878,750	25,463,625,000	99.57	0.02; 0.04	96.97; 92.87	92.68; 85.31	36.24; 36.51
RHC00873_L7	65,711,298	19,713,389,400	99.42	0.02; 0.04	97.97; 92.93	94.37; 84.94	36.09; 36.37
RHC00873_L4	98,597,863	29,579,358,900	99.68	0.02; 0.04	97.28; 93.27	93.68; 86.43	35.20; 35.31
RHC00873_L5	81,484,657	24,445,397,100	99.62	0.02; 0.04	97.23; 93.12	93.28; 85.89	35.12; 35.37

Note: * data of two groups represent the analysis results of reads sequenced two times.

Table S5. Summary statistics of the genome sequencing data of *C. sinensis*. Related to Figure 1.

Pair-end libraries	Insert size (bp)	Total data (Gb)	Read length (bp)	Sequence coverage (X)
Illumina reads	350	58.02	150	67.16
PacBio reads	-	103.29	-	119.56
10 × genomics	-	123.29	150	142.69
Hi-C	-	102.22	150	118.32
Total	-	386.81	-	447.73

Table S6. Transcriptome sequencing data of *C. sinensis*. Related to Figure 1.

Library ID	Sample	Raw reads	Clean reads	Clean bases (Gb)	Error rate (%)	Q20 (%)	Q30 (%)	GC (%)	rRNA rate (%)
RRA121485-V	Digestive gland	88,336,102	87,848,700	13.18	0.03	97.92	93.74	37.12	3.87
RRA121486-V	Gonad	60,295,788	59,970,826	9.00	0.03	97.59	93.00	35.67	1.40
RRA121487-V	Foot	67,290,820	66,624,456	10.00	0.03	97.55	92.85	34.86	11.20
RRA121488-V	Adductor muscle	71,259,096	70,680,622	10.60	0.03	97.84	93.68	38.16	3.60
RRA121489-V	Mantle	68,296,522	67,828,178	10.18	0.03	97.84	93.70	36.69	2.37
RRA121490-V	Pipe	71,082,130	70,403,448	10.56	0.03	97.64	93.10	36.10	3.70
RRA121491-V	Gill	72,441,054	71,822,822	10.78	0.03	97.86	93.55	35.49	3.87
Total		499,001,512	495,179,052	74.30	-	-	-	-	-
Average		-	-	-	0.03	97.75	93.37	36.30	4.29

Table S7. Summary statistics of the survey of the *C. sinensis* genome based on K-mer=17. Related to Figure 1.

K-mer	K-mer number	K-mer depth	Genome size (Mb)	Revised genome size (Mb)	Heterozygous ratio (%)	Repeat (%)
17	43,043,433,636	49	878.44	863.95	1.53	48.31

Table S8. Contig assembly of the *C. sinensis* genome. Related to Figure 1.

Title	Total length (bp)	Total number	Max length (bp)	Number (length≥2000 bp)	N50 length (bp)	N50 number (bp)	N90 length (bp)	N90 number (bp)
Contig*	1,408,901,898	1,652	-	1,645	2,013,216	219	507,293	747
Contig**	1,413,351,864	1,652	-	1,645	2,019,203	219	508,893	747
Contig***	902,806,104	594	7,948,157	-	2,626,413	114	907,036	324

Note: *refers to contigs assembled using PacBio data; **refers to contigs assembled after error correction; ***refers to contig assembly after heterozygosity reduction based on error-corrected contig assembly.

Table S9. Genome assembly of *C. sinensis* using Illumina and 10X Genomics. Related to Figure 1.

Title	Total length (bp)	Total number	Max length (bp)	N50 length (bp)	N50 number	N90 length (bp)	N90 number
Contig*	902,806,104	594	7,948,157	2,626,413	114	907,036	324
Scaffold*	903,895,197	441	11,906,054	3,588,323	83	1,319,859	240
Contig**	902,101,413	583	7,945,429	2,694,996	112	928,278	318
Scaffold**	903,120,697	441	11,893,072	3,586,861	83	1,318,971	240

Note: * refers to the genome assembly using data from PacBio and 10X Genomics; ** refers to the genome assembly after error correction using Illumina data based on forward-step genome assembly.

Table S10. Summary statistics of the *C. sinensis* genome assembly. Related to Figure 1.

	Length		Number	
	Contig* (bp)	Scaffold (bp)	Contig* (bp)	Scaffold (bp)
Total	902,101,413	903,158,897	701	187
Max	7,945,429	71,315,799	-	-
Num≥2000	-	-	689	183
N50	2,587,078	46,470,132	118	9
N60	2,183,475	44,700,546	155	11
N70	1,831,041	44,100,560	200	13
N80	1,351,472	43,035,416	258	15
N90	868,483	38,441,806	339	17

Note: only scaffolds greater than 100 bp in length were counted. N50 refers to the length of sequence equal to or greater than half of the total sequence length. * refers to contig after scaffolding.

Table S11. Assembly statistics of the published bivalve genomes. Related to Figure 1.

Species	Contig N50 (kb)	Scaffold N50 (kb)	Genome size (Gb)	Complete BUSCO (%)	Reference
Cyclina sinensis	2,587.1	46,470.1	0.90	92.7	In the present study
Crassostrea virginica	1,971.2	75,944.0	0.68	94.6	Gomez-Chiarri et al., 2015
Saccostrea glomerata	39.8	804.2	0.78	79.0	Powell et al., 2018
Mizuhopecten yessoensis	37.6	803.6	0.99	-	Wang et al., 2017
Limnoperna fortunei	-	312	1.67	81.9	Uliano-Silva et al., 2018
Chalmys farreri	21.5	602	0.78	91.9	Li et al., 2017
Modiolus philippinarum	19.7	100.2	2.38	82.1	Sun et al., 2017
Bathymodiolus platifrons	13.2	343.3	1.64	91.4	Sun et al., 2017
Argopecten purpuratus	80.1	1020	0.72	89.0	Li et al., 2018
Ruditapes philippinarum	28.1	345	1.12	92.2	Yan et al., 2019
Ruditapes philippinarum	13.0	48.4	2.56	-	Mun et al., 2017
Scapharca broughtonii	1,797.7	44,995.7	0.88	91.3	Bai et al., 2019
Sinonovacula constricta	976.9	65,929.7	1.22	91.9	Ran et al., 2019
Crassostrea gigas	19.4	401.3	0.56	-	Zhang et al., 2012
Pinctada fucata	1.6	14.5	1.15	-	Takeuchi et al., 2012

Table S12. Assessment of the genome coverage rate using raw reads. Related to Figure 1.

	Sample ID	Percentage
Reads	Mapping rate (%)	95.59
	Average sequencing depth	49.41
Genome	Coverage (%)	99.80
	Coverage at least 4X (%)	99.59

Note: mapping rate, the number of total reads that mapped to the assembled genome; average sequencing depth, the average sequencing depth that mapped to assembled genome; coverage, the sequence coverage of the assembled genome; coverage at least 4X, the coverage percentage of bases with depth >4X in whole genome bases.

Table S13. SNP results of the *C. sinensis* genome. Related to Figure 1.

Title	Number	Percentage (%)
All SNP	7,240,186	0.8128
Heterozygosis SNP	7,232,603	0.8120
Homology SNP	7,583	0.0008

Table S14. CEGMA results of the *C. sinensis* genome. Related to Figure 1.

Species	Complete		Partial	
	Prots	Completeness (%)	Prots	Completeness (%)
Cyclina sinensis	213	85.89	19	7.66

Note: CEGMA (Core Eukaryotic Genes Mapping Approach) defined the number of 248 ultraconserved CEGs that occur in a wide range of eukaryotes. A protein is classified as partial if the alignment of the predicted protein to the HMM profile represents less than 70% of the original KOG domain; otherwise, it is classified as complete.

Table S15. BUSCO results of the *C. sinensis* genome. Related to Figure 1.

BUSCO categories	Percentage
Complete	92.7%
Complete single-copy	91.6%
Complete duplicate	1.1%
Fragmented	1.3%
Missing BUSCOs	6.0%
Total BUSCO groups searched	978

Note: Completely, the lengths of the recovered matches were within the expectation of the BUSCO (benchmarking universal single-copy orthologs) profile match lengths. If these matches found only once were defined as ‘complete single-copy’, while more than once were defined as ‘complete duplicate’. The matches only partially recovered were defined as ‘Fragmented’, and BUSCO groups with no matches were defined as ‘Missing BUSCOs’.

Table S16. Prediction of repeat elements in the *C. sinensis* genome. Related to Figure 1.

Type	Repeat size (bp)	Percentage (%)
TRF	108,629,991	12.03
RepeatMasker	333,366,184	36.91
ProteinMask	40,582,418	4.49
Total	389,581,791	43.14

Note: the tandem repeats and interspersed repeats were predicted in the *C. sinensis* genome. The tandem repeats were predicted by TRF (Tandem repeats finder), and the interspersed repeats were predicted by RepeatMasker and ProteinMask.

Table S17. Categories of repeat elements predicted in the *C. sinensis* genome. Related to Figure 1.

		**Repeatmasker**		**TE proteins**		**Combined TEs**	
		**Length (bp)**	**% in genome**	**Length (bp)**	**% in genome**	**Length (bp)**	**% in genome**
DNA transposon	DNA	206,630,056	22.88	10,406,041	1.15	212,929,026	23.58
	LINE	35,570,247	3.94	20,325,648	2.25	47,194,345	5.23
Retrotransposon	SINE	2,555,960	0.28	0	0	2,555,960	0.28
	LTR	59,737,576	6.61	10,114,135	1.12	62,466,390	6.92
Other	Simple Repeat	5,795,835	0.64	0	0	5,795,835	0.64
	Unknown	46,174,425	5.11	0	0	46,174,425	5.11
	Total	333,366,184	36.91	40,582,418	4.49	349,664,813	38.72

Table S18. Statistics of noncoding RNA of the *C. sinensis* genome. Related to Figure 1.

	Type	Number	Average length (bp)	Total length (bp)	% of genome
miRNA		885	109.69	97,078	0.010749
tRNA		1,934	74.64	144,361	0.015984
rRNA	rRNA	35	102.91	3,602	0.000399
	18s	9	129	1,161	0.000129
	28s	2	116.5	233	0.000026
	5.8s	0	0	0	0
	5s	24	92	2,208	0.000244
snRNA	snRNA	239	134.99	32,263	0.003572
	CD-box	56	91.43	5,120	0.000567
	HACA-box	58	173.1	10,040	0.001112
	splicing	120	136.31	16,357	0.001811

Table S19. Prediction of gene structure in *C. sinensis* genomes. Related to Figure 1.

	Gene set	Number	Average transcript length (bp)	Average CDS length (bp)	Average exons per gene	Average exon length (bp)	Average intron length (bp)
Denovo	Augustus	32,897	9,853.75	1,400.36	6.4	218.86	1,565.91
	GlimmerHMM	119,194	6,529.31	548.15	3.26	168.37	2,651.53
	SNAP	53,267	13,907.98	703.77	5.64	124.73	2,844.32
	Geneid	171,825	3,283.63	495.06	2.9	170.96	1,470.97
	Genscan	28,343	20,552.90	1,576.49	6.8	232.00	3,274.47
Homolog*	Bpl	37,340	3,435.17	852.04	3.01	282.90	1,284.00
	Cfa	23,814	6,006.59	989.84	4.23	234.19	1,554.82
	Cgi	29,864	5,114.49	1,065.99	3.89	273.89	1,399.88
	Obi	21,081	4,787.79	885.1	3.65	242.79	1,475.24
	Pca	19,770	6,585.50	1,101.4	4.60	239.32	1,522.40
	Pye	30,225	5,169.59	1,066.38	3.92	272.31	1,407.12
	Hsa	10,185	7,029.69	1,051.54	4.98	211.36	1,503.88
	Bta	10,311	6,779.99	1,003.9	4.86	206.61	1,496.83
RNAseq	PASA	50,569	12,110.21	1,045.79	5.16	202.76	2,661.18
	Cufflinks	94,513	21,630.79	3,027.35	7.53	402.10	2,849.41
EVM		36,985	10,704.78	1,301.28	6.28	207.27	1,781.56
Pasa-update		36,654	10,886.81	1,318.65	6.32	208.49	1,796.88
Final set		27,564	12,897.87	1,471.11	7.42	198.14	1,778.63

Note: * Bpl, *Bathymodiolus platifrons*; Cfa, *Chalmys farreri*; Cgi, *Crassostrea gigas*; Obi, *Octopus bimaculoides*; Pca, *Pomacea canaliculata*; Pye, *Patinopecten yessoensis*; Hsa, *Homo sapiens*; Bta, *Bos Taurus*.

Table S20. Gene structure of genomes of *C. sinensis* and other homologous species. Related to Figure 1.

Species	Number	Average transcript length (bp)	Average SDS length (bp)	Average exons per gene	Average exon length (bp)	Average intron length (bp)
Bpl	33,584	9,783.48	1,114.81	5.24	212.81	2,045.16
Cfa	28,602	11,130.41	1,414.90	6.58	214.90	1,739.92
Cgi	28,397	7,302.44	1,483.73	7.57	196.09	886.13
Obi	15,842	35,365.61	1,547.02	8.01	193.08	4,822.66
Pca	21,131	10,258.41	1,644.48	9.17	179.43	1,054.97
Pye	24,521	16,344.93	1,660.85	8.11	204.68	2,063.98
Final Set	27,564	12,897.87	1,471.11	7.42	198.14	1,778.63

Note: Bpl, *B. platifrons*; Cfa, *C. farreri*; Cgi, *C. gigas*; Obi, *O. bimaculoides*; Pca, *P. canaliculata*; Pye, *P. yessoensis*.

Table S21. Functional annotation of the predicted protein-coding genes in the *C. sinensis* genome assembly. Related to Figure 1.

Title	Number	Percent (%)
Total	27,564	100
Swissprot	19,036	69.10
Nr	24,040	87.20
KEGG	18,773	68.10
InterPro	27,170	98.60
GO	24,906	90.40
Pfam	18,209	66.10
Annotated	27,344	99.20
Unannotated	220	0.80

Table S22. Protein-coding genes used for gene family clustering in each species. Related to Figure 2.

Full name	Gene number	Date resource
Cyclina sinensis	27,564	Obtained in this study
Lottia gigantea	23,526	GCF_000327385.1
Biomphalaria glabrata	24,031	GCA_000457365.1
Crassostrea virginica	34,264	GCF_002022765.2
Crassostrea gigas	27,264	GCF_000297895.1
Pinctada fucata martensii	28,041	Takeuchi et al., 2012
Bathymodiolus platifrons	33,384	https://datadryad.org/stash/dataset/doi:10.5061/dryad.h9942
Modiolus philippinarum	36,266	https://datadryad.org/stash/dataset/doi:10.5061/dryad.h9942
Patinopecten yessoensis	23,930	GCF_002113885.1
Chalmys farreri	27,984	Li et al., 2017
Branchiostoma floridae	28,407	GCF_000003815.1
Ruditapes philippinarum	27,652	Yan et al., 2019
Haliotis discus hannai	28,869	Nam et al., 2017
Homo sapiens	22,748	GCF_000001405.38

Table S23. GO enrichment of unique gene families in *C. sinensis* compared with 13 other species. Related to Figure 2.

GO ID	GO Term	GO Class	P-value	Adjusted P-value	Gene Number
GO:0008146	sulfotransferase activity	MF	1.73E-22	3.18E-19	47
GO:0008113	peptide-methionine (S)-S-oxide reductase activity	MF	9.87E-09	3.00E-06	8
GO:0001733	galactosylceramide sulfotransferase activity	MF	1.29E-07	2.64E-05	13
GO:0030246	carbohydrate binding	MF	4.80E-05	0.005517776	36
GO:0016667	oxidoreductase activity, acting on a sulfur group of donors	MF	7.14E-05	0.007723075	12
GO:0008080	N-acetyltransferase activity	MF	0.000474644	0.031190911	9
GO:0008970	phosphatidylcholine 1-acylhydrolase activity	MF	0.000597932	0.036673151	3
GO:0004963	follicle-stimulating hormone receptor activity	MF	0.001761263	0.067761796	6
GO:0007217	tachykinin receptor signaling pathway	BP	0.003263491	0.100080394	6
GO:0009404	toxin metabolic process	BP	0.007109476	0.162997155	3
GO:0016493	C-C chemokine receptor activity	MF	0.014647609	0.256681906	10
GO:0009066	aspartate family amino acid metabolic process	BP	0.015149693	0.261745985	6
GO:0004392	heme oxygenase (decyclizing) activity	MF	0.016160812	0.265341386	2
GO:0006788	heme oxidation	BP	0.016160812	0.265341386	2
GO:0004692	cGMP-dependent protein kinase activity	MF	0.018125686	0.269593852	5
GO:0051240	positive regulation of multicellular organismal	BP	0.019317253	0.282093225	8

	process				
GO:0005923	tight junction	CC	0.024806693	0.322018523	10
GO:0005165	neurotrophin receptor binding	MF	0.02597818	0.322018523	2
GO:0004066	asparagine synthase (glutamine-hydrolyzing) activity	MF	0.02597818	0.322018523	2
GO:0006529	asparagine biosynthetic process	BP	0.02597818	0.322018523	2
GO:0042891	antibiotic transport	BP	0.02642652	0.322018523	10
GO:0001607	neuromedin U receptor activity	MF	0.030379778	0.340846288	5
GO:0006108	malate metabolic process	BP	0.037575676	0.393977159	4
GO:0016615	malate dehydrogenase activity	MF	0.037575676	0.393977159	4
GO:0007586	digestion	BP	0.039278478	0.40602472	6

Table S24. KEGG enrichment of unique gene families in *C. sinensis* compared with 13 other species. Related to Figure 2.

Map ID	Map Title	P-value	Adjusted P-value	Gene Number
map00532	Glycosaminoglycan biosynthesis – chondroitin sulfate / dermatan sulfate	4.87E-18	9.39E-16	27
map04514	Cell adhesion molecules (CAMs)	1.89E-05	0.001723643	16
map04668	TNF signaling pathway	2.68E-05	0.001723643	15
map05200	Pathways in cancer	0.000130068	0.006275787	30
map00533	Glycosaminoglycan biosynthesis - keratan sulfate	0.000592532	0.022871751	8
map04640	Hematopoietic cell lineage	0.000895991	0.028821039	10
map05222	Small cell lung cancer	0.001307387	0.036046533	13
map05321	Inflammatory bowel disease (IBD)	0.00371927	0.079891179	4
map00720	Carbon fixation pathways in prokaryotes	0.003868841	0.079891179	5
map05206	MicroRNAs in cancer	0.004139439	0.079891179	20
map00040	Pentose and glucuronate interconversions	0.005252855	0.090438582	7
map04215	Apoptosis - multiple species	0.005623124	0.090438582	9
map00534	Glycosaminoglycan biosynthesis - heparan sulfate / heparin	0.007852837	0.113254493	5
map00965	Betalain biosynthesis	0.008215352	0.113254493	4
map00740	Riboflavin metabolism	0.009410592	0.121082947	4
map04075	Plant hormone signal transduction	0.014976049	0.178417089	3
map04320	Dorso-ventral axis formation	0.016439858	0.178417089	11
map00250	Alanine, aspartate and glutamate metabolism	0.017009609	0.178417089	6
map05145	Toxoplasmosis	0.019756512	0.190650342	13
map00051	Fructose and mannose metabolism	0.024665097	0.205164863	5

map00513	Various types of N-glycan biosynthesis	0.025439121	0.205164863	12
map04145	Phagosome	0.025512729	0.205164863	16
map04623	Cytosolic DNA-sensing pathway	0.026575902	0.205165966	7
map00020	Citrate cycle (TCA cycle)	0.03045351	0.217686204	5
map04742	Taste transduction	0.03045351	0.217686204	5
map00620	Pyruvate metabolism	0.035324314	0.24348545	6
map04977	Vitamin digestion and absorption	0.041339794	0.275123455	6
map00950	Isoquinoline alkaloid biosynthesis	0.044884544	0.288757233	4
map03430	Mismatch repair	0.048201907	0.295848375	4

Table S29. Summary of positively selected genes in two buried bivalves (*C. sinensis* and *R. philippinarum*). Related to Figure 1.

Gene ID	NR Annotation	Gene Abbreviation
Hic_asm_0.2081	ubiquitin carboxyl-terminal hydrolase 7-like isoform X3 [Crassostrea gigas]	*ucn7*
Hic_asm_7.697.1	sodium/potassium-transporting ATPase subunit beta-1-interacting protein 3-like isoform X1 [Crassostrea gigas]	*nkain3*
Hic_asm_11.770	uncharacterized protein LOC105345697 [Crassostrea gigas]	-
Hic_asm_1.1274	F-box/LRR-repeat protein 2-like [Crassostrea gigas]	*fbxl2*
Hic_asm_6.479	DNA repair protein complementing XP-G cells homolog [Crassostrea gigas]	-
Hic_asm_7.410	methenyltetrahydrofolate synthase domain-containing protein isoform X2 [Notothenia coriiceps]	*mthfsd*
Hic_asm_10.1586	alpha-actinin, sarcomeric-like isoform X1 [Crassostrea gigas]	*actn*
Hic_asm_2.1098	caprin-1-like isoform X2 [Crassostrea gigas]	*caprin-1*
Hic_asm_10.1123	protein YIPF4-like [Crassostrea gigas]	*yipf4*

Table S30. GO enrichment of positively selected genes in two buried bivalves (*C. sinensis* and *R. philippinarum*). Related to Figure 1.

GO ID	GO Term	GO Class	P-value	Adjusted P-value	Gene Number
GO:0007015	actin filament organization	BP	0.000343645	0.023759335	2
GO:0051017	actin filament bundle assembly	BP	0.000361359	0.023759335	1
GO:0051764	actin crosslink formation	BP	0.000361359	0.023759335	1
GO:0030272	5-formyltetrahydrofolate cyclo-ligase activity	MF	0.000722601	0.031674025	1
GO:0006996	organelle organization	BP	0.004159371	0.107591731	3
GO:0045033	peroxisome inheritance	BP	0.006845998	0.107591731	1
GO:0009396	folic acid-containing compound biosynthetic process	BP	0.008281941	0.107591731	1
GO:0005779	integral component of peroxisomal membrane	CC	0.008281941	0.107591731	1
GO:0005158	insulin receptor binding	MF	0.010790401	0.107591731	1
GO:0016337	single organismal cell-cell adhesion	BP	0.011506067	0.107591731	1
GO:0005884	actin filament	CC	0.01792638	0.113796591	1
GO:0004221	ubiquitin thiolesterase activity	MF	0.018992821	0.113796591	1
GO:0045010	actin nucleation	BP	0.021477181	0.113796591	1
GO:0006511	ubiquitin-dependent protein catabolic process	BP	0.028192277	0.117691567	1
GO:0006289	nucleotide-excision repair	BP	0.02960075	0.119769188	1
GO:0022607	cellular component assembly	BP	0.032452174	0.124414022	2

GO:0003697	single-stranded DNA binding	MF	0.038013551	0.131546894	1
GO:0016788	hydrolase activity, acting on ester bonds	MF	0.038863281	0.131704261	2
GO:0004519	endonuclease activity	MF	0.04323857	0.139794142	1

Table S31. KEGG enrichment of positively selected genes in two buried bivalves (*C. sinensis* and *R. philippinarum*). Related to Figure 1.

Map ID	Map Title	P-value	Adjusted P-value	Gene Number
map05203	Viral carcinogenesis	0.003290535	0.049358018	2
map00670	One carbon pool by folate	0.013231077	0.0987065	1
map05412	Arrhythmogenic right ventricular cardiomyopathy (ARVC)	0.025391319	0.0987065	1
map05322	Systemic lupus erythematosus	0.026321733	0.0987065	1
map03420	Nucleotide excision repair	0.035586851	0.106760553	1
map05146	Amoebiasis	0.047982742	0.107709052	1

Table S32. Enriched GO terms of expanded genes in the *C. sinensis*. Related to Figure 3.

GO ID	GO Term	GO Class	P-value	Adjusted P-value	Gene Number
GO:0006898	receptor-mediated endocytosis	BP	5.11E-41	3.63E-38	29
GO:0005044	scavenger receptor activity	MF	2.95E-29	5.25E-27	30
GO:0051258	protein polymerization	BP	1.59E-16	1.89E-14	16
GO:0034622	cellular macromolecular complex assembly	BP	4.74E-11	3.37E-09	21
GO:0006461	protein complex assembly	BP	2.60E-10	1.59E-08	23
GO:0007017	microtubule-based process	BP	4.47E-10	2.27E-08	18
GO:0005874	microtubule	CC	7.53E-10	3.57E-08	15
GO:1901565	organonitrogen compound catabolic process	BP	1.49E-08	4.70E-07	17
GO:0006184	GTP catabolic process	BP	1.84E-08	4.70E-07	15
GO:0005856	cytoskeleton	CC	2.76E-08	5.45E-07	20
GO:0015630	microtubule cytoskeleton	CC	2.90E-08	5.58E-07	18
GO:0003924	GTPase activity	MF	4.35E-08	8.14E-07	15
GO:0044450	microtubule organizing center part	CC	1.06E-07	1.75E-06	7
GO:0009056	catabolic process	BP	1.82E-07	2.82E-06	19
GO:0044248	cellular catabolic process	BP	2.24E-07	3.19E-06	18
GO:1901575	organic substance catabolic process	BP	6.67E-07	9.13E-06	18
GO:0044712	single-organism catabolic process	BP	6.85E-07	9.19E-06	18
GO:0044430	cytoskeletal part	CC	9.70E-07	1.19E-05	18
GO:0016043	cellular component organization	BP	1.80E-06	2.10E-05	24

GO:0015057	thrombin receptor activity	MF	8.87E-06	9.14E-05	7
GO:0070493	thrombin receptor signaling pathway	BP	8.87E-06	9.14E-05	7
GO:0000930	gamma-tubulin complex	CC	1.05E-05	0.000105571	5
GO:0031122	cytoplasmic microtubule organization	BP	1.05E-05	0.000105571	5
GO:0043232	intracellular non-membrane-bounded organelle	CC	1.11E-05	0.000109812	26
GO:0007020	microtubule nucleation	BP	1.19E-05	0.000113978	5
GO:0009055	electron carrier activity	MF	1.40E-05	0.000132909	11
GO:0005525	GTP binding	MF	2.41E-05	0.000211186	15
GO:0000226	microtubule cytoskeleton organization	BP	3.99E-05	0.000341662	9
GO:0043228	non-membrane-bounded organelle	CC	4.16E-05	0.000351731	28
GO:0009117	nucleotide metabolic process	BP	4.29E-05	0.000358945	16
GO:0044446	intracellular organelle part	CC	4.79E-05	0.000395605	24
GO:0044422	organelle part	CC	9.76E-05	0.000797445	26
GO:0020037	heme binding	MF	0.000494189	0.003660087	12
GO:0005506	iron ion binding	MF	0.000535187	0.003882839	12
GO:0007010	cytoskeleton organization	BP	0.000887356	0.006309103	10
GO:0000774	adenyl-nucleotide exchange factor activity	MF	0.000984581	0.006863106	2
GO:0042803	protein homodimerization activity	MF	0.001306781	0.008776655	2
GO:1901135	carbohydrate derivative metabolic process	BP	0.001365145	0.009071197	16
GO:0044424	intracellular part	CC	0.001860742	0.012137503	52
GO:0043229	intracellular organelle	CC	0.001912605	0.012362381	43
GO:0006996	organelle organization	BP	0.002237088	0.014201514	15

GO:0043226	organelle	CC	0.002290994	0.014415016	45
GO:0016705	oxidoreductase activity, acting on paired donors, with incorporation or reduction of molecular oxygen	MF	0.002329716	0.014530071	10
GO:0051087	chaperone binding	MF	0.002531936	0.015653971	2
GO:0044281	small molecule metabolic process	BP	0.003045639	0.018508114	21
GO:0016712	oxidoreductase activity, acting on paired donors, with incorporation or reduction of molecular oxygen, reduced flavin or flavoprotein as one donor, and incorporation of one atom of oxygen	MF	0.003470032	0.020732715	4
GO:0000242	pericentriolar material	CC	0.003558117	0.021081846	2
GO:0008792	arginine decarboxylase activity	MF	0.004132249	0.023886417	2
GO:0019887	protein kinase regulator activity	MF	0.004547802	0.025460528	4
GO:0008295	spermidine biosynthetic process	BP	0.004746298	0.025958598	2
GO:0043234	protein complex	CC	0.006368596	0.033541271	29
GO:0006527	arginine catabolic process	BP	0.006822215	0.035434485	2
GO:0004872	receptor activity	MF	0.006827742	0.035434485	53
GO:0004879	ligand-activated sequence-specific DNA binding RNA polymerase II transcription factor activity	MF	0.008108939	0.040601801	8
GO:0005952	cAMP-dependent protein kinase complex	CC	0.009228456	0.044982982	3

Table S33. Enriched KEGG pathways of expanded genes in the *C. sinensis*. Related to Figure 3.

Map ID	Map Title	P-value	Adjusted P-value	Gene Number
map05130	Pathogenic Escherichia coli infection	5.35E-18	2.46E-16	15
map04612	Antigen processing and presentation	3.75E-15	8.61E-14	12
map04540	Gap junction	2.05E-14	3.14E-13	15
map05169	Epstein-Barr virus infection	1.02E-13	1.17E-12	18
map04213	Longevity regulating pathway – multiple species	1.96E-13	1.80E-12	12
map05164	Influenza A	4.56E-13	3.49E-12	15
map05134	Legionellosis	2.51E-12	1.65E-11	12
map04210	Apoptosis	1.61E-11	9.26E-11	15
map04145	Phagosome	1.87E-11	9.56E-11	15
map05162	Measles	5.65E-11	2.60E-10	12
map05145	Toxoplasmosis	1.04E-09	4.35E-09	12
map04915	Estrogen signaling pathway	1.22E-09	4.68E-09	12
map04141	Protein processing in endoplasmic reticulum	3.53E-09	1.25E-08	12
map04144	Endocytosis	6.35E-09	2.09E-08	15
map03040	Spliceosome	1.20E-08	3.67E-08	12
map04010	MAPK signaling pathway	5.37E-08	1.54E-07	12
map04640	Hematopoietic cell lineage	1.30E-05	3.52E-05	6
map00140	Steroid hormone biosynthesis	1.69E-05	4.33E-05	5
map04917	Prolactin signaling pathway	3.60E-05	8.72E-05	5
map04913	Ovarian steroidogenesis	4.42E-05	0.00010155	5
map00590	Arachidonic acid metabolism	0.001396653	0.003059335	4

map05221	Acute myeloid leukemia	0.002756406	0.005763394	3

Table S39. List of tyrosinase family genes specific to two buried bivalves (*C. sinensis* and *R. philippinarum*). Related to Figure 4.

Gene ID	NR Annotation
evm.model.Hic_asm_17.791	Putative tyrosinase-like protein tyr-3 [*C. gigas*]
evm.model.Hic_asm_17.470	Putative tyrosinase-like protein tyr-3 [*C. gigas*]
evm.model.Hic_asm_18.1803	Putative tyrosinase-like protein tyr-3 [*C. gigas*]
evm.model.Hic_asm_18.1804	Putative tyrosinase-like protein tyr-3 [*C. gigas*]

Transparent Methods

1 *Cyclina sinensis* sampling and nucleic acid preparation

Healthy *Cyclina sinensis* samples were collected in Dandong, Liaoning Province, China. A 3-year-old female *C. sinensis* individual was sampled, dissected and frozen in liquid nitrogen immediately for DNA extraction. High-quality genomic DNA was extracted from the adductor muscle and gills of *C. sinensis* with a phenol-chloroform method (Green and Sambrook, 2012). The extracted DNA was measured using a Nanodrop 2000 (Thermo Scientific, USA) and a Qubit 2.0 (Invitrogen, USA) bioanalyzer system. Transcriptomic samples from different adult tissues (mantle, gonad, digestive gland, gill, adductor muscle, pipe and foot) of another 3-year-old individual were collected for mRNA library preparation. Total RNA was isolated using TRIzol reagent (Invitrogen, USA) according to the manufacturer's instructions. After the RNA was purified using an RNeasy Mini Kit (Qiagen), its quality was evaluated by the 28S/18S ratio and RNA integrity number (RIN) value using an Infinite F200 (TECAN, Switzerland) and Bioanalyzer 2100 system (Agilent Technologies, Santa Clara, CA).

2 Library construction and sequencing

For the short-read sequencing library, high-quality genomic DNA was sheared to ≈350 bp for Illumina HiSeq PE sequencing using the Covaris S2 Ultrasonicator system, and a short-read sequencing library was constructed using Illumina DNA library preparation kits according to standard protocols. A large-insert (30 kb) SMRTbell library was prepared using a 20 kb lower-end size selection protocol on BluePippin (Sage Science). The 350 bp DNA library was subjected to 100/150 bp sequencing on the Illumina HiSeq PE150 platform, and the 30 kb DNA library was subjected to SMRT sequencing (average read length >10 kb) on the PacBio Sequel platform (Pacific Biosciences). To prepare the 10X Genomics library, high-molecular

weight-genomic DNA fragments (> 50 kb) were precisely partitioned by adding a specific barcode sequence in oil droplets on the GemCode platform such that all fragments produced within a partition shared a common barcode, followed by sequencing library construction and sequencing on the Illumina HiSeq PE150 platform. High-throughput chromosome conformation capture (Hi-C) technology was applied for chromosome-scale scaffolding of the genome assembly, and the *in vitro* Hi-C library was prepared using mantle cells following standard protocols (Rao et al., 2014). In addition, general eukaryotic cDNA libraries were constructed using the NEB Next® Ultra™ RNA Library Prep Kit for Illumina® (NEB, USA) following the manufacturer's instructions for transcriptomic samples from different adult tissues and sequenced on the Illumina HiSeq PE150 platform (HiSeq X Ten).

3 Estimation of genome size and assembly

Prior to *C. sinensis* genome assembly, genome size and genome heterozygosity were estimated based on *k*-mer analysis. The primary contigs of the *C. sinensis* genome were assembled with Falcon (v0.7+git.3a3e5817959fbc05898c7ed7442c2b67e46e6934) using PacBio platform data under default parameters (Chin et al., 2013). The primary assembled contigs were error-corrected using PacBio platform data by Quiver (smrtlink_5.0.1; https://www.pacb.com/support/software-downloads/). To address the problem of significant genome heterozygosity, an iteration strategy was used for contig assembly of the *C. sinensis* genome by purge_haplotigs software (version 1.0.2+; https://bitbucket.org/mroachawri/purge_haplotigs/src/master/). After the above contig assembly procedures, error-corrected and high-quality assembled contigs were finally obtained. In addition, two assist assembly technologies were employed to produce the final assembled genome. During the assist assembly, two genome assembly versions were produced. Assembly v1 (contigs/scaffolds) was first produced by combining linked reads from the 10X Genomics platform with PacBio-assembled contigs using fragScaff software (version 140324; https://sourceforge.net/projects/fragscaff/files/),

and gap filling was performed with Pilon software (version 1.18; https://github.com/broadinstitute/pilon) using paired-end clean reads from the Illumina platform. The contact maps generated from the Hi-C platform were merged to assembly v1 to produce assembly v2 (contigs/chromosome-scale scaffolds) using Lachesis software (version 201701; https://github.com/shendurelab/LACHESIS), and the misassembled scaffolds were corrected using Juicebox v1.8 software (Robinson et al., 2018; https://github.com/aidenlab/Juicebox). The consistency of the final genome assembly was evaluated by single nucleotide polymorphism (SNP) analyses using SAMtools (http://samtools.sourceforge.net/), and the completeness of the final genome assembly was evaluated by the Core Eukaryotic Genes Mapping Approach (CEGMA, http://korflab.ucdavis.edu/dataseda/cegma/) using 248 core eukaryotic genes and Benchmarking Universal Single-Copy Orthologs (BUSCO v3.0, http://busco.ezlab.org/) analyses using 978 conserved metazoan genes with default settings (Parra et al., 2007; Waterhouse et al., 2018).

4 Genome annotation

4.1 Repeat identification

For repeat annotation, tandem repeats were predicted using the software Tandem Repeats Finder (Benson, 1999), and transposable elements (TEs) were predicted via two approaches, including *de novo*-based and homology-based approaches. The *de novo* repeat library was constructed using RepeatModeler v1.0.4 (http://www.repeatmasker.org) and integrated with Repbase (http://www.girinst.org/repbase). This integrated *de novo* repeat library was used for prediction using RepeatMasker (http://www.repeatmasker.org) (Tarailo-Graovac and Chen, 2009). The homology-based approach was performed to identify known TEs (including long and short interspersed elements, long terminal repeats, and DNA transposons) by aligning *C. sinensis* genome sequences against Repbase (nucleotide and protein library; http://www.girinst.org/repbase) using RepeatMasker and

RepeatProteinMask (both available on website: http://www.repeatmasker.org).

4.2 Noncoding RNA prediction

Noncoding RNA (ncRNA) genes, including transfer RNAs (tRNAs), ribosomal RNAs (rRNAs), microRNAs (miRNAs), and small nuclear RNAs (snRNAs), were predicted from the *de novo*-assembled *C. sinensis* genome using Infernal v1.1.2 software (Nawrocki and Eddy, 2013) by alignment with the Rfam ncRNA database (http://xfam.org/) under default parameters (Kalvari et al., 2018). In addition, the prediction of tRNA positions was also performed using tRNAscan-SE with default parameters (Lowe and Eddy, 1997).

4.3 Gene prediction and function annotation

The prediction of genes in the *C. sinensis* genome was performed using a combination of three approaches: homolog-based, *de novo*, and transcriptome-based predictions. For homolog-based gene prediction, nonredundant protein sequences from six species of mollusks (*Crassostrea gigas*, *Octopus bimaculoides*, *Bathymodiolus platifrons*, *Chlamys farreri*, *Pomacea canaliculata*, and *Patinopecten yessoensis*) and two species of mammals (*Homo sapiens* and *Bos taurus*) were aligned to the *C. sinensis* genome using tblastn (https://blast.ncbi.nlm.nih.gov) with an E-value cutoff of 1E-5 (Altschul et al., 1997), and the homologous genome sequences were aligned to the matched proteins using GeneWise v2.4.1 (http://www.ebi.ac.uk/~birney/wise2/) for accurate gene region prediction (Birney et al., 2004). For *de novo* gene prediction, the repeat-masked genome sequences of *C. sinensis* were used to predict gene structure using three gene prediction tools: Augustus v2.7 (http://bioinf.uni-greifswald.de/augustus/) (Keller et al., 2011), GlimmerHMM v3.02 (http://ccb.jhu.edu/software/glimmerhmm/) (Majoros et al., 2004) and SNAP v4.0 (http://snap.stanford.edu/snappy/index.html) (Leskovec and Sosič, 2016). The RNA-Seq data from different tissues (mantle, gonad, digestive gland, gill, adductor

muscle, pipe and foot) were aligned to the *C. sinensis* genome using TopHat v2.1.1 (Trapnell et al., 2009). The assembled transcripts were produced using Cufflinks v2.1.1 (Trapnell et al., 2012; Ghosh and Chan, 2016), and transcript structures were predicted. A consensus gene set for *C. sinensis* was produced with the three gene prediction methods (homology-based, *de novo*, and transcriptome-based) using EVidenceModeler (Haas et al., 2008), and the rank criterion of different sources was set as 'trans' > 'homog' > '*de novo*'. The gene prediction data from EVidenceModeler were modified by adding the annotations of untranslated regions (UTRs) and alternative splicing sites using PASA software (Haas et al., 2003), and a final gene set for *C. sinensis* was obtained. Gene functional annotation was performed by searching the SwissProt (http://www.uniprot.org/), NR (nonredundant protein, https://www.ncbi.nlm.nih.gov/protein), and KEGG (http://www.genome.jp/kegg/) databases using BLASTP v2.10 software, by searching the InterPro (https://www.ebi.ac.uk/interpro/) database using InterProScan v78.0 (https://github.com/ebi-pf-team/interproscan), and by alignment to the Pfam (https://pfam.xfam.org/) database using HMMER v3.3 software (http://hmmer.org/) and the GO (http://www.geneontology.org/) database using Blast2GO v5.2 software (https://www.blast2go.com/).

5 Gene family analysis

Gene families were defined for 14 selected species, including 12 mollusk species (*C. sinensis*, *Ruditapes philippinarum*, *Lottia gigantea*, *Biomphalaria glabrata*, *Crassostrea virginica*, *C. gigas*, *Pinctada fucata martensii*, *B. platifrons*, *Modiolus philippinarum*, *P. yessoensis*, *C. farreri*, and *Haliotis discus hannai*) and two representatives of chordates (*H. sapiens* and *Branchiostoma floridae*). Gene families were clustered among the selected species using OrthoMCL software (version 1.4) (Li et al., 2003). An all-against-all BLASTP analysis was used to determine the gene similarities between different genomes with a cutoff of 1e-7, and then a hierarchical clustering algorithm was applied to group orthologs and paralogs from all selected

species with an inflation value (-I) of 1.5. The longest transcript of each gene was retained, and the genes encoding polypeptides shorter than 30 amino acids were abandoned. Gene families presented in *C. sinensis* but not in any other species were regarded as *C. sinensis*-specific gene families.

6 Phylogeny, divergence time and evolutionary rate estimation

To investigate the phylogenetic relationships of the Venus clam with other species, a phylogenetic tree was reconstructed based on the shared single-copy gene families (only one gene copy in a gene family cluster for each species) retrieved from the above 14 selected species (*H. sapiens* and *B. floridae* were chosen as the outgroup species). The single-copy orthologous genes were aligned using MUSCLE (version 3.6) (Edgar, 2004) and concatenated to a super-alignment matrix. A maximum likelihood (ML) tree was built based on the super-alignment matrix using RAxML software (version 8.0.19) (Stamatakis et al., 2005). The best-fitting amino acid substitution model (LG + Γ4 model) was selected using the program ProtTest (ModelTest version 3.4) (Darriba et al., 2011), and the ML tree was assessed using the bootstrap method (1,000 bootstrap replicates). The divergence time between species/clades was estimated using the MCMCTree program implemented in PAML software with the following parameters: burn in=5,000,000, sample number=1,000,000, sample frequency=50 (Yang, 2007). Five reference divergence times for calibrations were retrieved from the TimeTree database (Kumar et al., 2017), including 484.9~482.4 million years ago (Mya) for *B. glabrata* and *H. hannai*, 511~520.1 Mya of *L. gigantea* and *C. gigas*, 208.9~361.7 Mya for *C. gigas* and *P. martensii*, 534.8~582.3 Mya for *B. floridae* and *M. philippinarum*, and 60.1~183.8 Mya for *M. philippinarum* and *B. platifrons*.

For substitution rate analysis, two buried bivalves (*C. sinensis* and *R. philippinarum*) were chosen as the foreground branch, and seven other sessile/semisessile bivalves (*C. virginica*, *C. gigas*, *P. martensii*, *C. farreri*, *P. yessoensis*, *M. philippinarum* and *B. platifrons*) were chosen as the background

branch. Multiple protein alignments from foreground and background branches were filtered by Gblocks v0.91b software (http://molevol.cmima.csic.es/castresana/Gblocks.html) to remove the low-quality aligned regions and then converted into the corresponding codon alignments for each gene family of the selected species (Castresana, 2000). The rate of nonsynonymous substitution (*Ka*, the number of nonsynonymous substitutions per nonsynonymous site) and the rate of synonymous substitution (*Ks*, the number of synonymous substitutions per synonymous site) were estimated using a branch-site model implemented in the PAML codeml program (http://abacus.gene.ucl.ac.uk/software/paml.html) (Yang, 2007). Comparison of *Ka* and *Ks* may reveal evidence that genes are under positive or negative selection (Zhang et al., 2006). If *Ks* is greater than *Ka*, this suggests that the gene is under negative selection, and to be stringent, only *Ks* values less than five were considered.

7 Expansion and contraction of gene families

For greater insight into the evolutionary dynamics of the genes, the expansion and contraction of the gene ortholog clusters were determined among the 14 species by comparing cluster sizes between ancestors and each current species using CAFE software (version 1.6) (De Bie et al., 2006). The gene gain and loss along each lineage of the RAxML tree were calculated by CAFE software with a random birth and death process model. A probabilistic graphical model (PGM) was introduced to calculate the probability of transitions in gene family size from parent to child on the phylogeny. The expanded and contracted gene families in *C. sinensis* were identified by comparison with other species, and expanded and contracted gene families in other species were identified by comparison with ancestors. KEGG and GO analyses were conducted based on gene families exclusively presented and specifically expanded and contracted in the buried bivalves (*C. sinensis* and *R. philippinarum*) using Blast2GO and KAAS (https://www.genome.jp/kegg/kaas/).

8 Karyotyping

Chromosomes were obtained with conventional methods (Duan et al., 2020). Gill tissue was dissected, soaked in 0.02% colchicine for 30 min, exposed to 0.075 M KCl solution for 40 min, fixed three times (each time for 20 min) with Carnoy's fixative (ethanol: glacial acetic acid = 3:1) and then dissociated into fine pieces by 50% acetic acid solution. Next, the resulting cell suspension was dropped onto a glass slide (56 ℃) and air dried. Finally, the cells were photographed and observed with a microscope, and karyotype analysis was performed with reference to Levan's standard (Levan et al., 1964).

9 Observation of fading in black shells

To investigate the fading of black shells, three treatment groups were arranged (10 individuals or 10 pairs of black shells from each group) in a pool without mud in well-lit room for observation. Live black-shelled clams were placed in cultured seawater (Group A). Black shells dissected from black-shelled clams were placed in cultured seawater (Group B) and air (Group C). White shells faded from black shells were placed in mud (group D). During the observation, five individuals or pairs of shells were randomly selected and photographed at 0, 14, 28, and 42 days. In addition, to observe the black color distribution in the shell, the black shells were cut using a mini cutting machine and observed and photographed under a stereo microscope.

10 Observation of melanin in black shells

Melanin was extracted from clam shells via hydrolysis in strong acids (Sun et al., 2017). The 100 g of shell powder obtained above was weighed and dissolved in 800 mL of 6 mol/L HCl solution. The HCl solution was discarded after the shell powder was sufficiently dissolved, and the residue was retained. To remove impurities, such as proteins, in the residue, the residue was placed in a round-bottomed flask, 800 mL of 6 mol/L HCl solution was added to it, and the flask was heated on a heating mantle

at 100 ℃ for 1 h. After sufficient reaction, the mixture was cooled and suction filtered, and the resulting residue was subjected to degreasing and drying. The rate of melanin extracted was calculated by the following formula: total amount of extracted melanin/total amount of sample. Ten milligrams of extracted black solid was sufficiently dissolved in 10 mL of 0.01 mol/L sodium hydroxide solution. UV spectroscopy was performed in the wavelength range of 190–500 nm using a UV spectrum scanner (UV-2550, Shimadzu, Japan), and other parameters were set to default. Sodium hydroxide solution (0.01 mol/L) served as the blank control, and three replicates were conducted in this assay.

Supplemental References

Altschul, S.F., Madden, T.L., Schäffer, A.A., Zhang, J., Zhang, Z., Miller, W., and Lipman, D.J. (1997). Gapped BLAST and PSI-BLAST: a new generation of protein database search programs. Nucleic Acids Res. 25(17), 3389–3402.

Bai, C.M., Xin, L.S., Rosani, U., Wu, B., Wang, Q.C., Duan, X.K., Liu, Z.H., and Wang, C.M. (2019). Chromosomal-level assembly of the blood clam, *Scapharca (Anadara) broughtonii*, using long sequence reads and Hi-C. Gigascience 8(7), pii: giz067.

Benson, G. (1999). Tandem repeats finder: a program to analyze DNA sequences. Nucleic Acids Res. 27(2), 573–580.

Birney, E., Clamp, M., and Durbin, R. (2004). GeneWise and Genomewise. Genome Res. 14(5), 988–995.

Castresana, J. (2000). Selection of conserved blocks from multiple alignments for their use in phylogenetic analysis. Mol. Biol. Evol. 17(4), 540-552.

Chin, C.S., Alexander, D.H., Marks, P., Klammer, A.A., Drake, J., Heiner, C., Clum, A., Copeland, A., Huddleston, J., Eichler, E.E., et al. (2013). Nonhybrid, finished microbial genome assemblies from long-read SMRT sequencing data. Nat. Methods 10(6), 563–569.

Darriba, D., Taboada, G.L., Doallo, R., and Posada, D. (2011). ProtTest 3: fast selection of best-fit models of protein evolution. Bioinformatics 27(8), 1164–1165.

De Bie, T., Cristianini, N., Demuth, J.P., and Hahn, M.W. (2006). CAFE: a computational tool for the study of gene family evolution. Bioinformatics 22(10), 1269–1271.

Duan, H.B., Chen, Y.H., Dong, Z.G., Zhang, M., Ge, H.X., Wei, M., Zhou, L.Q., Fu, X.C., and Sun, Z.P. (2020). Chromosome pand karyotype analysis of *Cyclina sinensis*. J. Fish. China 44(6): 1–7. (doi: 10.11964/jfc.20191012011)

http://kns.cnki.net/kcms/detail/31.1283.S.20200319.1247.004.html.

Edgar, R.C. (2004). MUSCLE: multiple sequence alignment with high accuracy and high throughput. Nucleic Acids Res. 32(5), 1792–1797.

Gómez-Chiarri, M., Warren, W.C., Guo, X., and Proestou, D. (2015). Developing tools for the study of molluscan immunity: The sequencing of the genome of the eastern oyster, *Crassostrea virginica*. Fish Shellfish Immunol. 46(1), 2-4.

Ghosh, S., and Chan, C.K. (2016). Analysis of RNA-Seq Data Using TopHat and Cufflinks. Methods Mol. Biol. 1374, 339–361.

Green, M., and Sambrook, J. (2012). Molecular Cloning: A Laboratory Manual. 4th Edition, Vol. II, (Cold Spring Harbor Laboratory Press).

Haas, B.J., Delcher, A.L., Mount, S.M., Wortman, J.R., Smith, R.K., Hannick, L.I., Maiti, R., Ronning, C.M., Rusch, D.B., Town, C.D., et al. (2003). Improving the Arabidopsis genome annotation using maximal transcript alignment assemblies. Nucleic Acids Res. 31(19), 5654–5666.

Haas, B.J., Salzberg, S.L., Zhu, W., Pertea, M., Allen, J.E., Orvis, J., White, O., Buell, C.R., and Wortman, J.R. (2008). Automated eukaryotic gene structure annotation using EVidenceModeler and the program to assemble spliced alignments. Genome Biol. 9(1), R7.

Kalvari, I., Argasinska, J., Quinones-Olvera, N., Nawrocki, E.P., Rivas, E., Eddy, S.R., Bateman, A., Finn, R.D., and Petrov, A.I. (2018). Rfam 13.0: shifting to a genome-centric resource for non-coding RNA families. Nucleic Acids Res. 46(D1), D335–D342.

Keller, O., Kollmar, M., Stanke, M., and Waack, S. (2011). A novel hybrid gene prediction method employing protein multiple sequence alignments. Bioinformatics 27(6), 757–763.

Kumar, S., Stecher, G., Suleski, M., and Hedges, S.B. (2017). TimeTree: a resource for timelines, timetrees, and divergence times. Mol. Biol. Evol. 34(7), 1812–1819.

Leskovec, J., and Sosič, R. (2016). SNAP: a general purpose network analysis and graph mining library. ACM Trans. Intell. Syst. Technol. 8(1), 1.

Levan, A., Fredga, K., and Sandberg, A.A. (1964). Nomenclature for centromeric position on chromosomes. Hereditas 52(2): 201–220. https://doi.org/10.1111/j.1601-5223.1964.tb01953.x

Li, C., Liu, X., Liu, B., Ma, B., Liu, F., Liu, G., Shi, Q., and Wang, C. (2018). Draft genome of the Peruvian scallop *Argopecten purpuratus*. Gigascience 7(4).

Li, L., Stoeckert, C.J., and Roos, D.S. (2003). OrthoMCL: identification of ortholog groups for eukaryotic genomes. Genome Res. 13(9), 2178–2189.

Li, Y., Sun, X., Hu, X., Xun, X., Zhang, J., Guo, X., Jiao, W., Zhang, L., Liu, W., Wang, J., et al. (2017). Scallop genome reveals molecular adaptations to semi-sessile life and neurotoxins. Nat. Commun. 8(1), 1721.

Lowe, T.M., and Eddy, S.R. (1997). tRNAscan-SE: a program for improved detection of transfer RNA genes in genomic sequence. Nucleic Acids Res. 25(5), 955–964.

Majoros, W.H., Pertea, M., and Salzberg, S.L. (2004). TigrScan and GlimmerHMM: two open source ab initio eukaryotic gene-finders. Bioinformatics 20(16), 2878–2879.

Mun, S., Kim, Y.J., Markkandan, K., Shin, W., Oh, S., Woo, J., Yoo, J., An, H., and Han, K. (2017). The Whole-genome and transcriptome of the manila clam (*Ruditapes philippinarum*). Genome Biol. Evol. 9(6), 1487–1498.

Nam, B.H., Kwak, W., Kim, Y.O., Kim, D.G., Kong, H.J., Kim, W.J., Kang, J.H., Park, J.Y., An, C.M., Moon, J.Y., et al. (2017). Genome sequence of pacific abalone (*Haliotis discus hannai*): the first draft genome in family Haliotidae. Gigascience 6(5), 1-8.

Nawrocki, E.P., and Eddy, S.R. (2013). Infernal 1.1: 100-fold faster RNA homology searches. Bioinformatics 29(22), 2933–2935.

Parra, G., Bradnam, K., and Korf, I. (2007). CEGMA: A pipeline to accurately annotate core genes in eukaryotic genomes. Bioinformatics 23(9), 1061–1067.

Powell, D., Subramanian, S., Suwansa-Ard, S., Zhao, M., O'Connor, W., Raftos, D., and Elizur, A. (2018). The genome of the oyster Saccostrea offers insight into the environmental resilience of bivalves. DNA Res. 25(6), 655–665.

Ran, Z., Li, Z., Yan, X., Liao, K., Kong, F., Zhang, L., Cao, J., Zhou, C., Zhu, P., He, S., et al. (2019). Chromosome-level genome assembly of the razor clam *Sinonovacula constricta* (Lamarck, 1818). Mol. Ecol. Resour. 19(6), 1647–1658.

Rao, S.S., Huntley, M.H., Durand, N.C., Stamenova, E.K., Bochkov, I.D., Robinson, J.T., Sanborn, A.L., Machol, I., Omer, A.D., Lander, E.S., et al. (2014). A 3D map of the human genome at kilobase resolution reveals principles of chromatin looping. Cell 159(7), 1665–1680.

Robinson, J.T., Turner, D., Durand, N.C., Thorvaldsdóttir, H., Mesirov, J.P., and Aiden E.L. (2018). Juicebox.js provides a cloud-based visualization system for Hi-C data. Cell Syst. 6(2), 256–258.

Stamatakis, A., Ludwig, T., and Meier, H. (2005). RAxML-III: a fast program for maximum likelihood-based inference of large phylogenetic trees. Bioinformatics 21(4), 456–463.

Sun, J., Zhang, Y., Xu, T., Zhang, Y., Mu, H., Zhang, Y., Lan, Y., Fields, C.J., Hui, J.H.L., Zhang, W., et al. (2017). Adaptation to deep-sea chemosynthetic environments as revealed by mussel genomes. Nat. Ecol. Evol. 1(5), 121.

Sun, X.J., Biao, W., Zhou, L.Q., Liu, Z.H., Dong, Y.H., and Yang, A.G. (2017). Isolation and characterization of melanin pigment from yesso scallop *patinopecten yessoensis*. J. Ocean Univ. China 16, 279–284.

Takeuchi, T., Kawashima, T., Koyanagi, R., Gyoja, F., Tanaka, M., Ikuta, T., Shoguchi, E., Fujiwara, M., Shinzato, C., Hisata, K., et al. (2012). Draft genome of the pearl oyster *Pinctada fucata*: a platform for understanding bivalve biology. DNA Res. 19(2), 117–130.

Tarailo-Graovac, M., and Chen, N. (2009). Using RepeatMasker to identify repetitive elements in genomic sequences. Curr. Protoc. Bioinformatics 25(1),

4.10.1–4.10.14. https://doi.org/10.1002/0471250953.bi0410s25.

Trapnell, C., Pachter, L., and Salzberg, S.L. (2009). TopHat: discovering splice junctions with RNA-Seq. Bioinformatics 25(9), 1105–1111.

Trapnell, C., Roberts, A., Goff, L., Pertea, G., Kim, D., Kelley, D.R., Pimentel, H., Salzberg, S.L., Rinn, J.L., and Pachter, L. (2012). Differential gene and transcript expression analysis of RNA-seq experiments with TopHat and Cufflinks. Nat. Protoc. 7(3), 562–578.

Uliano-Silva, M., Dondero, F., Dan Otto, T., Costa, I., Lima, N.C.B., Americo, J.A., Mazzoni, C.J., Prosdocimi, F., and Rebelo, M.F. (2018). A hybrid-hierarchical genome assembly strategy to sequence the invasive golden mussel, *Limnoperna fortunei*. Gigascience 7(2).

Wang, S., Zhang, J., Jiao, W., Li, J., Xun, X., Sun, Y., Guo, X., Huan, P., Dong, B., Zhang L, et al. (2017). Scallop genome provides insights into evolution of bilaterian karyotype and development. Nat. Ecol. Evol. 1(5), 120.

Waterhouse, R.M., Seppey, M., Simão, F.A., Manni, M., Ioannidis, P., Klioutchnikov, G., Kriventseva, E.V., and Zdobnov, E.M. (2018). BUSCO applications from quality assessments to gene prediction and phylogenomics. Mol. Biol. Evol. 35(3), 543–548.

Yan, X., Nie, H., Huo, Z., Ding, J., Li, Z., Yan, L., Jiang, L., Mu, Z., Wang, H., Meng, X., et al. (2019). Clam genome sequence clarifies the molecular basis of its benthic adaptation and extraordinary shell color diversity. iScience 19, 1225–1237.

Yang, Z. (2007). PAML 4: phylogenetic analysis by maximum likelihood. Mol. Biol. Evol. 24(8), 1586–1591.

Zhang, G., Fang, X., Guo, X., Li, L., Luo, R., Xu, F., Yang, P., Zhang, L., Wang, X., Qi, H., et al. (2012). The oyster genome reveals stress adaptation and complexity of shell formation. Nature 490(7418), 49–54.

Zhang, Z., Li, J., Zhao, X.Q., Wang, J., Wong, G.K., and Yu, J. (2006).

KaKs_Calculator: calculating Ka and Ks through model selection and model averaging. Genomics Proteomics Bioinformatics 4(4), 259–263.
